# Direct vs 2-stage approaches to structured motif finding

**DOI:** 10.1186/1748-7188-7-20

**Published:** 2012-08-21

**Authors:** Maria Federico, Mauro Leoncini, Manuela Montangero, Paolo Valente

**Affiliations:** 1 Dipartimento di Scienze Fisiche, Informatiche e Matematiche, Università di Modena e Reggio Emilia, 41125 Modena, Via Campi 213/b, Italy; 2 Istituto di Informatica e Telematica, Consiglio Nazionale delle Ricerche, 56124 Pisa, Via Moruzzi, 1, Italy

**Keywords:** Structured motif, TFBS discovery, Combinatorial algorithms

## Abstract

**Background:**

The notion of *DNA motif* is a mathematical abstraction used to model regions of the DNA (known as *Transcription Factor Binding Sites*, or *TFBSs*) that are bound by a given Transcription Factor to regulate gene expression or repression. In turn, *DNA structured motifs* are a mathematical counterpart that models sets of TFBSs that work in concert in the gene regulations processes of higher eukaryotic organisms. Typically, a structured motif is composed of an ordered set of isolated (or *simple*) motifs, separated by a variable, but somewhat constrained number of “irrelevant” base-pairs. Discovering structured motifs in a set of DNA sequences is a computationally hard problem that has been addressed by a number of authors using either a direct approach, or via the preliminary identification and successive combination of simple motifs.

**Results:**

We describe a computational tool, named SISMA, for the de-novo discovery of structured motifs in a set of DNA sequences. SISMA is an exact, enumerative algorithm, meaning that it finds all the motifs conforming to the specifications. It does so in two stages: first it discovers all the possible component simple motifs, then combines them in a way that respects the given constraints. We developed SISMA mainly with the aim of understanding the potential benefits of such a 2-stage approach w.r.t. direct methods. In fact, no 2-stage software was available for the general problem of structured motif discovery, but only a few tools that solved restricted versions of the problem. We evaluated SISMA against other published tools on a comprehensive benchmark made of both synthetic and real biological datasets. In a significant number of cases, SISMA outperformed the competitors, exhibiting a good performance also in most of the cases in which it was inferior.

**Conclusions:**

A reflection on the results obtained lead us to conclude that a 2-stage approach can be implemented with many advantages over direct approaches. Some of these have to do with greater modularity, ease of parallelization, and the possibility to perform adaptive searches of structured motifs. As another consideration, we noted that most hard instances for SISMA were easy to detect in advance. In these cases one may initially opt for a direct method; or, as a viable alternative in most laboratories, one could run both direct and 2-stage tools in parallel, halting the computations when the first halts.

## Background

Understanding the complex mechanisms that regulate gene expression is a pivotal problem in molecular biology. Gene transcription [1] starts when one or more regulatory proteins bind DNA regulatory elements, which are mostly located in the promoter region nearby the transcription start site (TSS) of genes, or also further apart in eukaryotic organisms (e.g. enhancers, silencers). In eukaryotes, DNA binding proteins are called transcription factors (TFs) and regulatory elements, to which they bind, are known as transcription factor binding sites (TFBSs).

In lower eukaryotes TFBSs are usually short DNA strings (5-25 base pairs long) bound by a single TF, that frequently appear, with possibly some mutations, upstream of the TSS in the proximal promoter region of co-regulated genes.

In higher eukaryotic organisms, transcription regulation is more complex and TFBSs are more difficult to characterize [2]. There may be multiple binding sites for a single TF in a single gene’s promoter region; there can be great variability in the binding sites of a single TF; the regulatory elements may be located also several kilobases away from the TSS, either upstream or downstream or in the introns of the genes that they regulate [3], and in this case they are often organized in functional groups (called cis-regulatory modules) [2] bound by several interacting TFs in a cooperative or antagonistic way.

Being able to identify TFBSs is crucial to our understanding of the mechanisms that regulate gene expression (e.g., chronology and cell-specificity of transcription [4]), and of the functions of individual genes regulated by newly discovered TFBSs [2]. Also, mutations in TFBS underlie several degenerative human diseases (e.g., all forms of cancer) and constitute a substantial component of the phenotypic variability within and across species [5].

Structural and functional information on mechanisms of interaction between TFs and their binding sites are provided by experimental techniques, which are costly and time-consuming.

### TFBS discovery as an algorithmic problem

The identification of (possible) functional sites can be formulated as an algorithmic problem, provided a mathematical abstraction is given to model TFBSs. Two of the most popular such models are *Position Specific Score Matrices* (*PSSM*) and *Hamming distance* (*HD*) models (see, e.g., [6,7]). Here we will adopt the HD model, which we now briefly recall.

**Figure 1:**
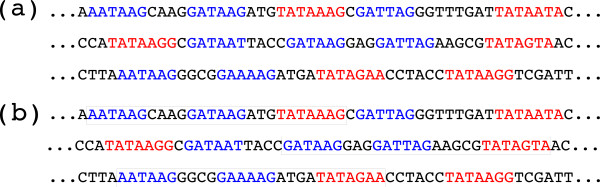
**Simple and structured motifs.** Part (**a**): some DNA sequences with instances of the two simple motifs GATAAG (one base substitution tolerated) and TATAAAA (up two base substitutions tolerated), highlighted in blue and red, respectively. Part (**b**): three instances of a structured motif (6,1)-[2,4]-(6,1)-[3,5] -(7,2).

In the HD model, a *simple motif M*_*w*_is given by a word *w* (over the DNA alphabet), sometimes called the *consensus*, together with an integer *e*, 0 ≤* e *< |*w*|. The *occurrences* of *M*_*w *_are those words *v* whose Hamming distance from the consensus is bounded by *e*, i.e., *d*_*H*_(*w*,*v*) ≤* e*. Note that *e *> 0 accounts for possible mutations (here only nucleotide substitutions) in functional sites relative to the same TF. Figure 1a shows examples of simple motifs and simple motif occurrences.

Computational *motif discovery* (or *motif finding*) can be defined as the task of inferring the mathematical abstractions subject to the identification of the occurrences (i.e., the potential binding sites) in the input sequences. The typical input to a motif discovery program under the HD model includes a pair (*ℓ*,*e*), which describes the length of the consensus and the maximum substitutions allowed in the occurrences, respectively.

Motif discovery is a very difficult problem [8], since the space of possible occurrences may be huge. The inverse problem, i.e., finding the occurrences given a motif definition, is called *motif search* and is instead comparatively much easier than discovery.

Simple motifs are typically used to represent TFBSs in lower eukaryotes. When more than one binding site is involved in gene regulation, as in higher eukaryotes, their collective formal description is more elaborate. Here, we are interested in formal models of so-called *structured motifs*, which can be simply defined as sets of simple motifs, often called *boxes*, whose occurrences, in the input DNA fragments, must satisfy given order and distance constraints. The input to a structured motif finder can be succinctly described using *template strings*:

$$\begin{array}{l} (\ell_{1},e_{1})-[d_{1},D_{1}]-(\ell_{2},e_{2})-\ldots \\ \;\;-(\ell_{b-1},e_{b-1})-[d_{b-1},D_{b-1}]-(\ell_{b},e_{b}) \end{array}$$

where, for all admissible *j* and *k*, *ℓ*_*j *_and *e*_*j *_constrain the motifs that can occur as box *j* according to the HD model, while *d*_*k*_ and *D*_*k *_are lower and upper bounds on the number of nucleotides between box *k* and box *k* + 1. Figure 1b illustrates the concept in case of *b *= 3.

### Focus of the paper

There is an already huge literature on motif discovery (see, e.g., [6,8-19] and also the references contained in the survey papers [20,21]). However, for our purposes the proposed algorithmic solutions fall into two classes: (1) optimization algorithms (either deterministic or probabilistic), and (2) enumerative, exact algorithms. Algorithms of the first class seek motifs that optimize a certain scoring function, usually exploring only a limited portion of the space of all possible motif candidates (see [6,9,10,22] for influential works). On the other hand, enumerative algorithms exhaustively search the motif space^a^[12,14,16,23,24].

A fundamental component of exact methods is what we can term the *enumeration engine*, i.e., the algorithm adopted to generate all the possible candidate motifs to be later evaluated on some statistical basis (for another example, see [25]). Actually, some exact motif finding tools have been proposed which are just enumeration engines, simply returning all the motifs that satisfy the input constraints [7,26-28]. Clearly, an appraisal of any such engine depends on its computational efficiency only.

The availability of enumeration tools is useful both because they can be taken as building blocks for more sophisticated finders and because they inspired (and still inspire) research in the whole field of exact methods. In this respect, it is worth observing that the tool which best behaved in the now famous assessment by Tompa et el. [29], namely Weeder, borrows its enumeration engines from [7].

In this paper we concentrate our attention on enumerative algorithms for structured motif discovery in a set of input DNA fragments. In particular, we focus on enumeration engines and base our analysis on running time and (to a lesser extent) memory consumption. We pay no attention to the “quality” of the results, simply because the output only depends on the input constraints posed to the motifs being sought. Running time is instead especially critical since faster enumeration leaves more room for post processing (i.e., picking the motifs deemed more likely to represent functional sites).

Before describing the contribution of our work, we analyze in more details the results presented in the literature that deal with enumeration engines for structured motif discovery.

### Related work

Existing algorithms are essentially based on one of two possible approaches: (1) directly explore the search space of structured motifs, or (2) first extract the simple motifs that may occur as boxes (using any available simple motif finder) and then “assemble” them into structured motifs that satisfy box order and distance constraints. We shall refer to the latter as to the *2-stage approach*.

A well-known potential advantage of directly exploring the space of structured motifs is that the combined boxes, together with the distance constraints, may be strong enough to quickly emerge, possibly together with few others spurious structures, even though each single box is a weak signal (see, e.g., [15]). We point out that the most efficient direct approach algorithms makes use of the (generalized) suffix tree data structure [30,31].

The 2-stage approach was first mentioned by Marsan and Sagot [32], who nonetheless deemed it impractical due to the high resource consumption. Recently, however, it was re-considered by Zhou et al. [28], who provided much tighter theoretical upper bounds on the runtime and space complexity. They designed the Ecomp algorithm and showed it to be more efficient than more sophisticated exact methods in their experimental settings.

Some available exact motif finders require that at least one instance of the motif be exact, *i.e.*, that it actually appears in one of the input sequences. This leads to a reduction of the motif search space with ensuing time and space savings. This simplified version of the problem is called *Frequent (Structured) Motif Discovery* problem [26].

#### SMILE, RISO and RISOTTO

SMILE [32] is a family of algorithms designed to solve slightly different variants of the structured motif discovery problem on set of input sequences. SMILE extends to structured motifs the algorithmic ideas on simple motif enumeration presented in [7]^b^. To explore the space of possible structured motifs, SMILE uses a generalized suffix tree of the input sequences together with a (virtual) lexicographic tree of all possible simple motifs. Improvements to SMILE are presented by Carvalho et al. [33]. Their Riso algorithm exhibits an exponential time and space gain over SMILE in the worst case. Riso works on a variation of the generalized suffix tree (called generalized factor tree) [34], built only up to the box length level, with some extra information used for fast update of the tree. Riso’s computational complexity is exponential with respect to the number of boxes and their lengths, but it does not depend on inter-box distances, thanks to the use of box links.

A further improvement is achieved by Risotto[27], although only on the average, thanks to it’s ability to quickly detect dead ends (i.e., words that cannot possibly be extended to a valid motif). In practice, Risotto is more than twice faster than Riso and, to the best of our knowledge, also the most efficient algorithm for exact enumeration of structured motifs composed of any number of boxes. For this reason, we will take Risotto as our primary competitor in the experimental tests.

#### ECOMP

Ecomp is a general, 2-stage algorithm that uses Mitra-count [15] to find all simple motifs and the starting positions of all their occurrences^c^. In the second step, the algorithm looks for dyads by checking all pairs of occurrences of simple motifs, keeping and counting only those satisfying the distance constraint. At the end, Ecomp outputs only the dyads satisfying the quorum constraint.

Unfortunately, Ecomp is fully described and tested only for dyads and source code is apparently not available.

#### ExMotif

The 2-stage approach is also used by ExMotif[26] to solve the frequent structured motif extraction problem. ExMotif’s main data structures are lists that store the positions of patterns appearing in the sequences. These lists are repeatedly intersected in order to find motifs that satisfy the input constraints. The output produced by ExMotif includes the structured motifs satisfying the quorum constraint and only the positions of their exact occurrences. The computational cost of ExMotif, as reported in [26], is exponential with respect to the number and the length of boxes.

#### Results

Our contribution is twofold. We present a novel 2-stage algorithm, called SISMA, that enumerates all the structured motifs conforming to input specifications. More precisely, we describe two different software tools that implement SISMA’s ideas. The first version, called SISMA_Smile, solve the “unconstrained” enumeration problem, while the second one, named SISMA_Speller, addresses the frequent structured motif discovery problem. We compare the performances of SISMA_Smile (resp., SISMA_Speller) against those of Risotto (resp. ExMotif) on a comprehensive dataset composed of both synthetic and real biological data. The experimental results show that our tools are competitive in enumerating spaces of structured motif candidates.

We also try to go one step further and reflect on the relative merits of direct vs 2-stage approaches for structured motifs finding. The latter enjoy some potential design advantages, such as modularity and ease of parallelization (see the concluding section for more on these aspects). However, the argument of computational inefficiency has often been used to discourage their active use. From our experiments here we can not devise strong arguments in favor of any of the two approaches. It is true that, in some circumstances, direct methods can explore spaces which are beyond the capabilities of a 2-stage algorithm. However, in other cases our 2-stage approach software results much faster than the competitor direct tool. In the concluding section we will give some guidelines (depending on parameter sets) to possibly assist the users to choose the most suitable tool for the problem instances at hand.

## Methods

In this section we describe the implementation of *SISMA* (“Successive Intersection of Simple Motifs Apart”), a structured motif finder based on the 2-stage approach.

SISMA is an exact algorithm which takes in **input** the following set of parameters: 

1. the set of sequences, in Fasta format, where the motifs must be found;

2. the number *b *≥ 2 of boxes (simple motifs) which the structured motifs will be made of;

3. an ordered set of *b* pairs of integers: (*ℓ*_*i*_,*e*_*i*_), *i *= 1,…,*b*, such that *ℓ*_*i *_is the length of the *i*^th^ box and *e*_*i *_the corresponding number of admissible errors;

4. for each pair of consecutive boxes, say the *i*^th^ and *i* + 1^th^ ones, a pair of integers (*d*_*i*_,*D*_*i*_) that specify the minimum and maximum number of bases, respectively, that may separate the two boxes, *i *= 1,…,*b *− 1.

5. a value *q *∈ (0,1] (the so-called *quorum*) that specifies the minimum fraction of input sequences that must contain an instance for the structured motif to be considered valid.

The **output** of the algorithm is made of all the possible structured motifs that conform to input specifications.

SISMA is implemented in C++ and its source code is available for download from http://algo.ing.unimo.it/mf/.

### Basic implementation

SISMA stores simple and structured motifs using vector data structures that make it possible to perform list intersections and filtering operations (i.e., the distance and quorum checks described below) in a very efficient way (technical details can be found in Additional file 1).

In current implementations, SISMA comes in two versions, which will be referred to as SISMA_Smile and SISMA_Speller, solving the structured and the frequent motif discovery problems, respectively.

#### Stage 1

SISMA_Smile first calls SMILE [32] for simple motif discovery. To the best of our knowledge, SMILE is the only tool available for download which is exact and that returns the positions of all the occurrences of found motifs^d^.

SISMA_Speller uses our implementation of the Speller[7] algorithm, which returns simple motifs with at least one exact match in the input sequences, together with all their occurrences. We decided for a new Speller implementation because, apparently, there is no available tool with these characteristics^e^.

Independently of the simple motif finder adopted, the output of the first stage is a set of simple motifs with associated position lists of their occurrences, each one ordered by increasing sequence indexes and increasing positions within the sequence. Logically, motifs returned by stage 1 are classified into *b* subsets, denoted by *K*_*i*_,*i *= 1,…,*b*, such that *m *∈* K*_*i*_ if and only if *m* can be the *i*^th^ box of one of the structured motifs being sought. Each set *K*_*i*_is maintained as a vector data structure, each cell of which in turns stores a pointer to a vector containing all occurrences of exactly one *m *∈* K*_*i*_, ordered by increasing input sequence index and increasing position in each sequence.

Observe that there is no filtering process of simple motifs found in this stage, because there is apparently no relations between significance of simple motifs and significance (or mere existence) of structured motif, as clearly stated in [35]. In particular, structured motifs might exist (and reach quorum) only because they contain weak simple motifs.

#### Stage 2

For *i *= 0,…,*b*, we will use the term *i-prefix* to denote any structured motif made of *i* boxes that could possibly be extended to a full structured motif conforming to the problem specification.

In the second stage, which is divided in *b* steps, SISMA builds prefixes of increasing length, starting from the empty prefix in step 1 and ending with *b*-prefixes (*i.e.*, full structured motifs) in step *b*. Basically, at generic step *i*, SISMA considers all (*i *− 1)-prefixes *p* and all motifs *m *∈* K*_*i*_ to assemble possible *i*-prefixes *r *=* p *− (*d*_*i*_,*D*_*i*_) −* m*. The computed *i*-prefixes are stored in a vector data structure, analogously to what is done with simple motifs.

During a prefix assembly step, SISMA checks distance and quorum constraints, in order to discard, as early as possible, prefixes that could not possibly be extended to full structured motifs.

*Distance check:* for each potential *i*-prefix *r* being built, *r *=* p *− (*d*_*i*_,*D*_*i*_) −* m*, and any sequence *s*, SISMA performs a binary search on the sorted occurrences of *m* in *s* in order to find the first occurrence that satisfies the minimum distance constraint. Then, all the subsequent occurrences of *m* are considered, until one is found that violates the maximum distance constraint. In this way, SISMA builds all occurrences of *r*.

*Quorum check:* the *i*-prefix *r* is discarded if its occurrences appear in less than *q*|*S*| input sequences. In fact, it is obvious that prefix extension can only reduce the eventual motif quorum.

### Options

The basic implementation has been enhanced with some options that might be used to have even more efficient second stage runs, under some circumstances. Implementation details can be found in Additional file 1.

#### Box index selection option

When this option is selected, SISMA builds structured motifs by considering boxes of increasing total number of occurrences and not by increasing index order. In more pictorial terms, the full (final) structured motifs are not determined by assembling longer and longer prefixes but rather longer and longer structured motif “subsequences”.

When using this option, before starting the *b* steps of stage 2, SISMA computes the total number of simple motif occurrences in each set *K*_*i*_, *i *= 1,…,*b*:

(1) $$B_{i}=\underset{t:m_{t}\in K_{i}}{\sum }|\text{oc}c_{i,t}|,$$

where *occ*_*i*,*t*_ is the set of occurrences of motif *m*_*t *_∈* K*_*i*_, *t *= 1,…,|*K*_*i*_|. SISMA then sorts the sets {*B*_*i*_}_*i *= 1,…,*b*_ and forms a list *L* with the corresponding box indexes, i.e., if *L*_*i *_=* j *and *L*_*i* + 1_ =* k*, then *B*_*j *_≤* B*_*k*_, *i *= 1,…,*b *− 1. Then, it uses the list *L* in stage 2 to determine the order with which to add the boxes to the structured motifs being built.

This option allows to limit the number of *useless* intermediate structured motifs (*i*-prefixes) that are generated (*i.e.*, those that eventually would be discarded, because they either could not be extended to full *b*-boxes or would not satisfy the quorum constraint). This is particularly effective when the expected number of structured motifs in the output is not large, which is likely to happen, *e.g.*, when the box length is large and a small number of errors are admitted, or simply when the number of boxes is large. There is a trade off between the slowdown introduced to store and handle extra information needed to implement this option and the speedup obtained by reducing the number of useless intermediate structured motifs. If the output is large, the slowdown is predominant; in contrast, if the output is small, speedup is predominant.

In practice, this option helped SISMA to drastically reduce the out-of-memory failures, especially on synthetic data.

#### Space-saving option

Sometimes the output to be generated is very large. This happens, *e.g.*, when the first stage has returned a huge number of simple motifs occurrences. In turns, this can be a consequence of the particular values of the input parameters, such as very short motif lengths and/or relatively small difference between length and number of available errors. Under these circumstances, SISMA basic implementation, which keeps all the intermediate motifs (including the simple ones) in main memory, may fail due to memory shortage.

To cope with this situation, especially on low memory PCs, SISMA can be run with a specific option that produces the output in distinct slices, and that requires less main memory to produce the output of each slice. Given an integer *v* as the value of the space-saving option parameter, for *i *= 1,…,*b*, each set of motifs *K*_*i*_ is partitioned into ⌈|*K*_*i*_|/*v*⌉ subsets $K_{i}=\{K_{i,1},K_{i,2},\ldots ,K_{i,\lceil |K_{i}|/v\rceil }\}$, each one containing at most *v* distinct motifs. The second stage is then run once for each possible element in the Cartesian product $K_{1}\times K_{2}\times \cdots \times K_{b}$. In this way SISMA drastically reduces the number of intermediate structured motifs generated and usually avoids out-of-memory failures at the price of a moderate slowdown (see the Computational cost section).

The only cases that this variant of SISMA is not able to handle are those in which the memory shortage is due to stage 1, i.e., when the number of simple motifs (and their occurrences) is simply too large to fit in memory (we will see that this happens in few very difficult instances on synthetic data).

#### Occurrence print option

SISMA might be instructed to output the starting positions of all occurrences of the discovered structured motifs.

Not using this option allows a fair comparison with tools that do not print all motif occurrences, but just the motif definitions.

#### Computational cost

The time computational cost of SISMA is given by the cost of simple motif extraction plus that of occurrence list intersections. Here we will first refer to the basic version of SISMA, with only briefly mentioning the various options at the end of the section.

With the current implementation, the simple motif extraction tool must be run once for each different single box template (i.e., for all different (*ℓ*,*e*) pairs). Both SMILE and our modified version of Speller have worst-case time complexity in *O*(*Nt*_*ℓ*_*ν*(*e*,*ℓ*)), where *t*_*ℓ*_ is the number of suffix tree nodes at depth *ℓ*, *N* is the number of input sequences, and *ν*(*e*,*ℓ*) is the number of words of length *ℓ* that differ in at most *e* letters from a word *m* of length *ℓ*. It holds that *ν*(*e*,*ℓ*) ≤* ℓ*^*e*^|Σ|^*e*^. Hence, the time complexity is linear in the input size, but possibly exponential in the number *e* of substitutions. Thus, as we are working with the DNA alphabet, the first stage takes $O(N\cdot \overset{b}{\underset{i=1}{\sum }}t_{\ell_{i}}\ell_{i}^{e_{i}}4^{e_{i}})$.

Let *B*_*i*_be the total number of occurrences of simple motifs found for the *i*^*th*^ box in the first phase (see Equation 1), for *i *= 1,…,*b*, and let *S*_*j*_ be the total number of *j*-prefixes occurrences found during the *j*^*th*^ step of the second stage, for *j *= 2,…,*b*−1. The cost of the occurrence list intersection phase is upper bounded by:

$$\begin{array}{l} B_{1}\cdot B_{2}+S_{2}\cdot B_{3}+\cdots \\ \;\;\;\;\cdots +S_{b-1}\cdot B_{b}=\overset{b-1}{\underset{i=1}{\sum }}S_{i}\cdot B_{i+1} \end{array}$$

where equality holds since *S*_1_ =* B*_1_. Hence, the computational cost of SISMA is

(2) $$O\left(N\overset{b}{\underset{i=1}{\sum }}t_{\ell_{i}}\ell_{i}^{e_{i}}{\left|\Sigma \right|}^{e_{i}}+\overset{b-1}{\underset{i=1}{\sum }}S_{i}\cdot B_{i+1}\right).$$

Equation (2) clearly shows that the running time of the second stage depends essentially on the number of occurrences of simple motifs and that of intermediate structured motifs. Note, however, that if there is a large number of simple motifs the cost of first stage is high as well. Low cost of the first stage and high cost of second stage are possible only if there are relatively few simple motifs but many intermediate structured motifs. This is in principle possible, but in practice it hardly happens due to order and distance constraints, as the computational experiments clearly indicate. In practice, thus, the cost of extracting structured motifs is comparable with that of simple motif finding, at least when the starting positions of all the occurrences are required. As for the memory space used, it is not difficult to see that this is the maximum between: (1) SMILE space complexity needed to generate all the motifs occurrences for each box, and (2) the space needed to generate *j*-prefixes occurrences, *i.e.,O*(max_*j*∈[2*.b*−1]_{*S*_*j*_ + *B*_*j* + 1_}).

We now briefly consider options. As for index selection, it can be easily seen that the handling of more complex data structures in main memory introduces, in the worst case, time and memory penalties linear with the number *b* of boxes. In practice, however, non-worst case instances might run much faster with this option activated (see Options section). For what concerns the space saving option, it can be proved that the slowdown is constant, although the exact figures depend on low level implementation issues. On the machine used to perform the experiments, the running times with space saving activated were almost four times higher. We must point out, however, that the intended use of this option is just to avoid out-of-memory failures, and these can be regarded as infinite time computations. Then, in these cases the option can be thought to provide (sometimes) “unbounded” speedups. In this case, space requirement is dominated only by SMILE space requirement. Finally, SISMA actually generates all occurrences during computation, and hence printing them takes only linear time in the number of occurrences.

## Results

We have performed a series of computational experiments on both synthetic and real biological data with a twofold goal: (1) compare the direct and 2-stage approaches using the best available algorithm (Risotto) for the former and our SISMA_Smile code for the latter; (2) compare SISMA_Speller with ExMotif[26], the only exact tool for the frequent structured motif extraction problem which adopts the 2-stage approach and whose code is available (see the Related work section)^f^.

We performed all the experiments on an uniprocessor AMD Athlon 64 3200+ with 1GB of RAM, forcing a timeout of twelve hours for the execution of each tool.

### Tests on synthetic data

In this section we report the results of tests performed on synthetic data, which are often used to validate the effectiveness of existing methods in a fully controlled experimental setting, and to experimentally evaluate their scalability properties. In particular, we generated synthetic data sets according to the so called *Planted Motif Problem* (*PMP*) [36] in the following way:

**Sequence generation:** we randomly generated the sequences of the input set *S*, assuming the characters of each sequence be i.i.d. and with equal probability (0.25) assigned to each symbol. According to [36], the data sets contain 20 sequences of 600 characters each.

**Structured motif planting:** we selected the number *b* of boxes and the *b* pairs (*ℓ*_*i*_,*e*_*i*_) using different rules, which we will specify when describing the experiments. We generated distance constraints at random, making sure that the total maximum distance between the first and last box fit into the sequences. For each pair (*ℓ*_*i*_,*e*_*i*_), defining the template for a simple motif *m*_*i*_, *i *= 1,…,*b*, we first selected a random word *w*_*i*_(the “exact” instance of *m*_*i*_) and then generated |*S*| occurrences of *m*_*i*_, at Hamming distance ≤* e*_*i *_from *w*_*i*_, by substituting *e*_*i *_characters of *w*_*i *_with characters from Σ chosen uniformly at random. Finally, we planted the occurrences, one per sequence, by respecting box order and distance constraints (but otherwise at random). When generating a dataset to be tested using ExMotif, we planted at least one exact structured motif occurrence.

The parameters used to built the dataset where then used to run the motif finding tools on that dataset. The quorum is set to *q *= 1.0 in all tests.

The data set generating process outlined above produces boxes that are instances of the PMP. There is a wide literature on the PMP, especially for single motif extraction (see, e.g. [12,37,38]). Preliminary results relative to the structured motif extraction settings can be found in [39] and, limited to dyads, in [15,28]. Using a simple model [37], we can estimate the number *E*(*ℓe*) of simple motifs that one expects to find in a randomly generated sequence, depending on the length of the motif and on the number of allowed errors. Expectation of pairs makes some instances easier to solve than others. When talking about “difficult” instances we will refer to ones in which the expected number of randomly found motifs is high.

**Experimental settings:** Here we present results concerning a set of tests in which we planted boxes with variable lengths and number of allowed substitutions, randomly chosen among those with expectation close to one (*i.e.* for which the planted motif and a little number of other random motifs can be expected), over the following pairs: (9, 2), (10, 2), (11,2), (11, 3), (12, 3), (13, 3), (14, 1), (14, 2), (14, 3), (14, 4), (15, 1), (15, 2), (15, 3), (15, 4), (15, 5).

We varied the number *b* of boxes between 2 and 10 and ran the algorithms 20 times on different datasets. We ran SISMA with the *box index selection* option, which resulted very effective in this set of experiments.

Further results on synthetic data are briefly reported at the end of this section and, in details, in Additional file 2.

**Results and discussion:** To compare pairs of tools, we used two different measures: (1) *win-count*, i.e., given a common value of *b*, the number of times one tool outperformed the other; (2) *running times*: we report best, worst and average running times, as well as standard deviations, for each value of *b* used^g^ (we computed means and standard deviations omitting the best and worst time runs). Moreover, we separately computed the above measures by considering either all runs, or only runs where both tools ended computations.

### SISMA_Smile vs Risotto

**Figure 2:**
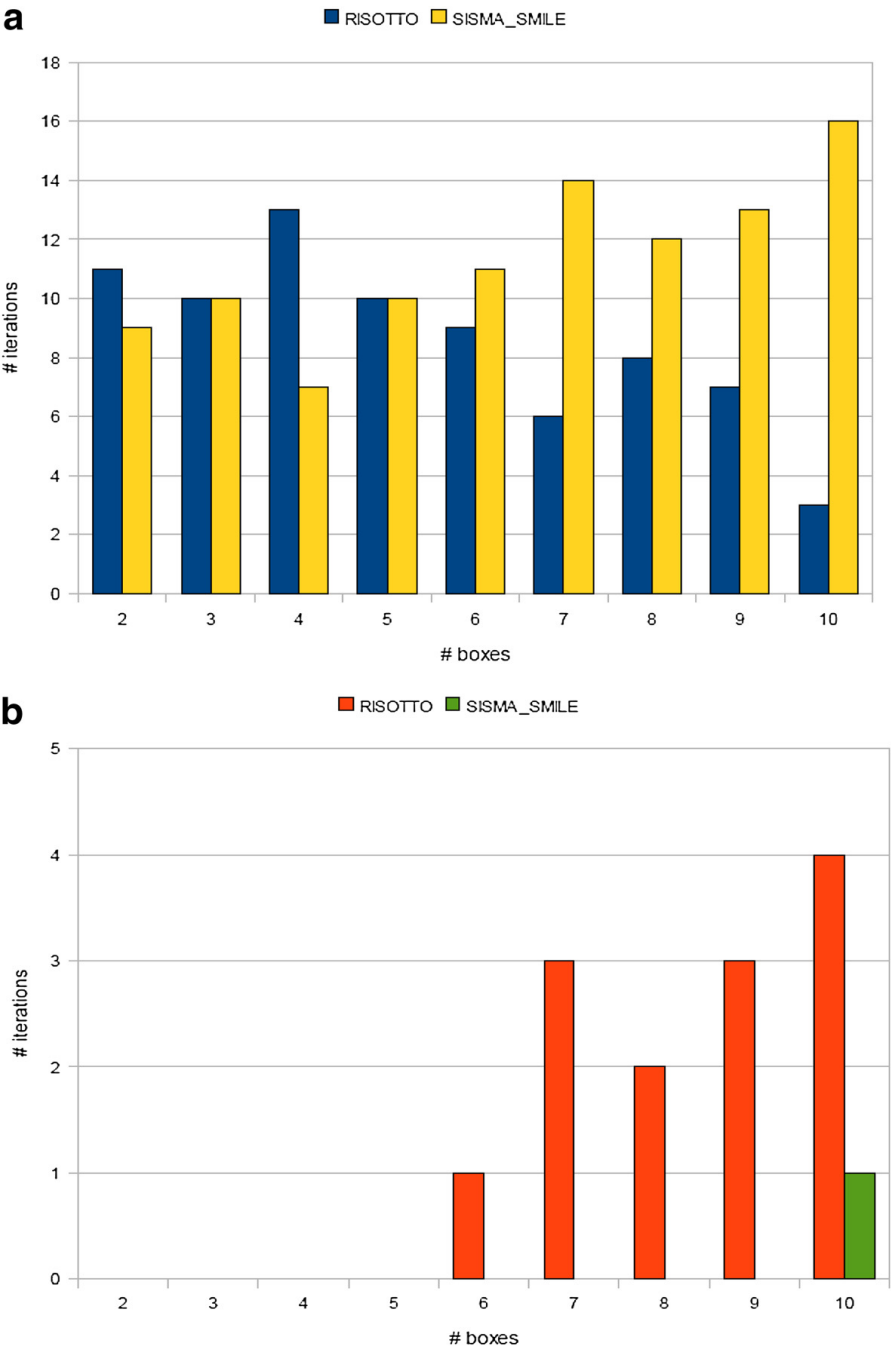
**Risotto vs SISMA_Smile on synthetic datasets (a) Number of iterations in which SISMA_Smile outperforms Risotto or vice versa, and (**b**) number of tools failures.** Notice that when one tool fails, the other might end computation, hence, failures might not sum up to the total number of runs.

Figure 2a reports win-counts, while Figure 2b reports the number of times each tool fails for particular values of *b*.

From Figure 2a we can see that no tool is definitely better than the other. Risotto is usually more competitive for small number of boxes (up to six), but turns significantly less competitive as the number of boxes increases. Moreover, Risotto failed on few runs even with relatively few (i.e., six or more) boxes, usually when the first boxes are hard instances of the PMP.

On the other hand, SISMA_Smile was almost as good as Risotto for small number of boxes while it handled larger problem instances definitively better. In the majority of tests, SISMA_Smile ended computation before Risotto and it failed only once due to memory shortage in the first stage (because of too many simple motif occurrences).

**Figure 3:**
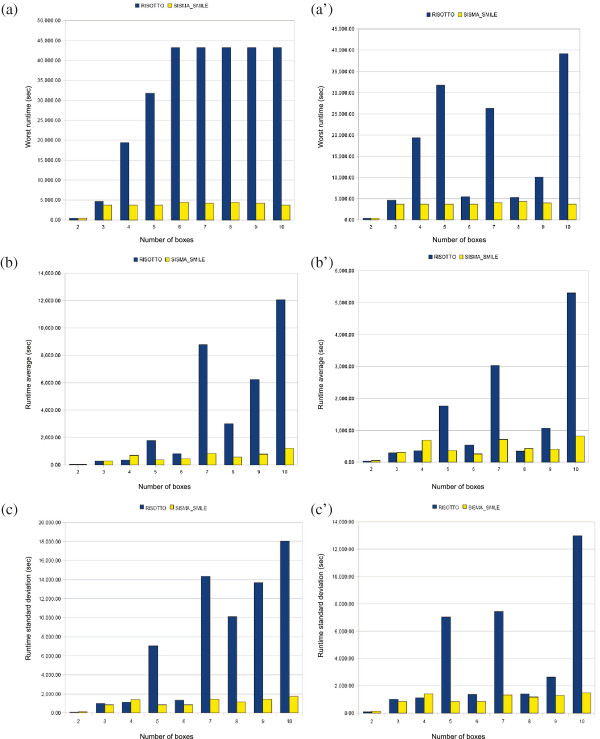
**Risotto’s and SISMA_Smile’s running times on synthetic datasets.** Worst and average run times and standard deviations (in seconds) for SISMA_Smile and Risotto. Average runtime and standard deviations have been computed omitting best and worst runs. (**a**) (**b**) (**c**) Running times of all runs are considered. (**a’**) (**b’**) (**c’**) Only running times of runs in which both tools end computation are considered. Notice that the scale on Y-axes is not the same for all charts.

Figure 3 reports running times. Note that, since Risotto is the tool which failed more frequently^h^, its charts show a greater difference in running times compared to SISMA_Smile’s. This means that the runs that were somehow difficult for Risotto were not particularly hard for SISMA_Smile. It also means, on the contrary, that SISMA_Smile’s failures occurred quite early during the computation (essentially as early as the length of a typical successful computation).

The best case was almost always favorable to Risotto, and there was no difference for what concerns best case when considering all runs or only those without failures. In the best case, Risotto ended computation in less than 10 seconds (with the exception of ten boxes, where the best run took 24 secs), while Risotto ended computation in less than 6 seconds for *b *≤ 5 and in about 30 seconds for *b *> 5, never exceeding 35 seconds.

Looking at Figure 3, we observe what follows: 

(a) In the worst case, SISMA_Smile is much faster than Risotto: the longer run for SISMA_Smile took less than 74*min*, while for Risotto some runs took more than 10*h*, even for relatively small number of boxes and excluding failures. Hence, even when Risotto defeated SISMA_Smile, the latter was still relatively fast. The opposite was not always true.

(b) Although average running times should be analyzed with care, Risotto showed average running times much worse than SISMA_Smile’s, even without considering failures. For instance, in case of ten boxes Risotto’s average runtime was around 1*h*and a half, while SISMA_Smile took less than 25min.

(c) Risotto showed a greater variance across all these data.

**Figure 4:**
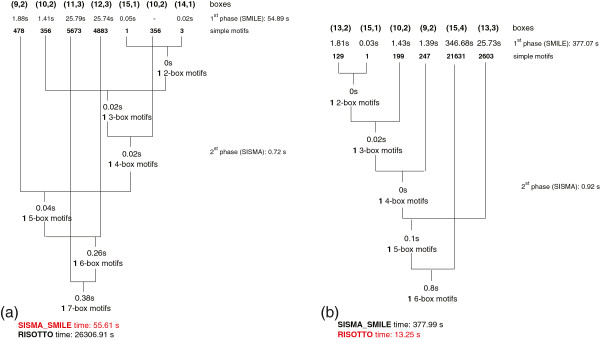
**Examples: SISMA_Smile vs Risotto.** Running times of Risotto, SISMA_Smile’s stages 1 and 2, and of all SISMA_Smile’s list intersection step (during stage 2). A 0s time for list intersection means that the corresponding step took time smaller than timer resolution. The box index selection order during stage 2 is shown. In example (**a**) SISMA_Smile outperforms Risotto. Observe that SMILE is called once on the (10,2) pair, so that the time reported for the 6^*th*^ box is 0. In this example Risotto is 473 times slower than SISMA. In example (**b**) Risotto outperforms SISMA_Smile because the stage 1 performed by SMILE is slow due to the presence of a box for which a large number of simple motifs is found. In this case SISMA_Smile is 28 times slower than Risotto. In particular, the most time consuming task is the extraction of the (15,4) box (about 91.7*%*of total execution time), for which 21631 simple motifs are found.

We further investigated the structure of the instances in which one tool outperformed the other in order to better understand advantages and disadvantages that may be typical of the direct and 2-stage approaches (see an example in Figure 4). We anticipate that the key factor is the first stage of SISMA_Smile. 

• SISMA_Smile outperforms Risotto when SISMA_Smile first stage is fast. This happens mainly for two reasons (that might happen simultaneously): (i) there is a small total number of simple motifs and SMILE running time is low. (ii) Boxes are characterized by the same pair (length, errors), and hence SMILE is run only once for each pair.

• Risotto*outperforms SISMA_*Smile when the first stage of simple motif extraction is slow due to boxes producing a large number of simple motifs. This situation might also affect SISMA_Smile second stage: a large number of intermediate structured motifs means a time consuming occurrence list intersection stage. In some cases the phenomenon is almost completely eliminated using the box index selection option.

Finally, a closer inspection on Risotto’s behavior shows that its running time may be highly affected by the positions of boxes with large search spaces, *e.g.*, box (14,4). We performed a set of experiments in which we searched for planted long structured motifs characterized by the same boxes (number and positions), with just one “floating” (14,4) box, which we moved from first to last position. While the details can be found in Additional file 2, we observe here that Risotto’s performance degraded considerably, while SISMA_Smile’s behavior was essentially unaffected by the (14,4) box position.

### SISMA_Speller vs ExMotif

We have no results for the comparison of SISMA_Speller and ExMotif, because the latter never ended computation within this experimental settings.

We explain this negative behavior observing that the set of pairs (*ℓ*,*e*) among which we chose the boxes of planted structured motifs contained several pairs characterized by large values of *ℓ* and *e*, which ExMotif is apparently not able to address.

On the other hand, SISMA_Speller never failed within this experimental setting, exhibiting low running times. The worst case run (even for ten boxes) was never above three minutes while the best case runs lasted around three seconds for two boxes and about 33 seconds in case of ten boxes.

#### Results for other synthetic experiments

we conclude this section by mentioning the results obtained for two other synthetic data sets (the details can be found in Additional file 2). 

• We tested the tools on “presumably” (i.e., à priori) easy instances for SISMA, where the structured motifs sought were composed by boxes of the same type (i.e., same length and number of errors). Indeed SISMA_Smile always outperformed Risotto when both tools ended computation, while ExMotif outperformed SISMA_Speller on input instances with very small values of *ℓ*,*e*, and *b*. For larger values, however, ExMotif did not end computations, while SISMA_Speller failed only when boxes were any of the known very hard PMP instances.

• The other data set was composed of presumably very hard instances, according to the PMP classification. We observed a relatively large number of failures, both of SISMA_Smile and Risotto. The reasons were essentially those already observed, but interestingly enough, the two tools did not (usually) fail on the same instances, meaning that a difficult instance for one tool might not be so difficult for the other. ExMotif did end computation only on a limited number of instances and only in very few cases it outperformed SISMA_Speller.

### Tests on real biological data

In this section we report the results of experiments performed on three different datasets composed of upstream regions of co-regulated genes of the *Saccharomyces cerevisiae* in order to extract motifs representing transcription factor binding sites.

#### UASH-URS1-10 dataset

The dataset was drawn from [13]. It contains the upstream sequences of 11 meiotic genes of the *Saccharomyces cerevisiae* which are cooperatively regulated by the transcription factors URS1H and UASH involved in the meiotic expression during sporulation.

These 11 genes are listed in SCPD [40]. In 10 out of the 11 genes, the URS1H binding site appears downstream from UASH site and both sites are located within the upstream region -300 to -1. We included these ten regions in the dataset. We do not included a sequence for the remaining gene (HOP1) since there the binding sites are reversed and the URS1H site is placed much further upstream compared to all the other genes in the set.

We designed the same experimental settings as in [26], except for the distance gap between the two sites. We chose a larger gap range with respect to [26] in order to approach the problem in a more realistic way, in which information about the binding site being sought may not be known. We look for structured motifs of the form (3,1)-[1,1]-(5,2)-[1,200]-(9,1). We required that structured motifs occurred in at least 7 sequences (quorum *q *= 70*%*). SISMA was run with the *space-saving* option.

#### UASH-URS1-5 dataset

In the 10 genes dataset discussed above, the two binding sites occur within at most 200 bases. However, as GuhaThakurta and Stormo suggest in [13], that gene sequences can be equally divided in two groups based on the average distance between UASH and URS1H sites. According to this, we obtained a group of 5 genes in which the binding sites are within 50 bases of each other.

We reproduced the experimental setup defined in [28]: we analyzed the five sequences in UASH-URS1-5 and looked for dyad motifs of the form (7,1)-[1,50]-(10,2) (again, we actually used a larger distance gap with respect to [28] in order to approach the problem in a more realistic way). Quorum was set to 80%, *i.e.*, at least 4 sequences.

#### KAR4P dataset

This dataset contains 23 genes of the *Saccharomyces cerevisiae* which are co-regulated by the KAR4p transcription factor required for gene regulation in response to pheromones. We obtained the list of 23 co-regulated genes from the YEASTRACT database [41] and the upstream regions of those genes using the RSAT [42] retrieve sequence tool.

We deduced the characteristics of the KAR4p binding site from the consensus given in YEASTRACT and we looked for structured motifs of the form (3,1)-[2,2]-(4,1)-[2,2]-(3,1)-[1,1]-(2,1) occurring in at least 68% of the input sequences, i.e., in at least 16 sequences.

#### Discussion

**Figure 5:**
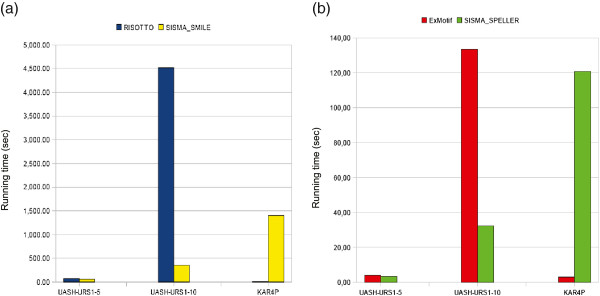
**Running times on biological datasets.** Running times (in seconds) of (**a**) SISMA_Smile and Risotto (**b**) SISMA_Speller and ExMotif on biological datasets, when using an uniprocessor machine with 1GB of RAM.

Figure 5 reports the running times (in seconds) for all the four tools, while Table 1 reports the number of simple and structured motifs found for each dataset and the average number of occurrences. We can make the following general observations: 

• ExMotif terminated computations in all the experiments, contrary to what happened on synthetic datasets. Here, structured motifs are composed of few boxes with few substitutions allowed, experimental conditions extremely favorable to ExMotif.

• Stage 2 is predominant on SISMA’s running time (see Table 1), in contrast with what we observed on the synthetic datasets, because here (except for UASH-URS1-5) there is a large number of occurrences for each box, making occurrence lists intersection a demanding task.

• SISMA’s stage 2 is characterized by running times which increase with the number of boxes and with the total number of occurrences, coherently with the theoretical bound (see the Methods section).

**Table 1:** Number of simple and structured motifs on biological datasets

**UASH-URS1_5**			
		SISMA_smile	SISMA_speller
**Box** (7,1)		1,452 (∼10)	420 (∼10)
**Box** (10,2)		5,472 (∼5)	92 (∼5)
**Structured Motif**			
(7,1) - [1,50] - (10,2)		16,662 (∼5)	14 (∼5)
**UASH-URS1_10**			
		SISMA_smile	SISMA_speller
**Box** (3,1)		64 (∼1000)	64 (∼1000)
**Box** (5,2)		1,024 (∼1000)	942 (∼1000)
**Box** (9,1)		103 (∼10)	55 (∼10)
**Structured Motif**			
(3,1) - [ 1,1 ] - (5,2) - [ 1,200 ] - (9,1)		2,309,173 (∼ 70)	7,241 (∼ 70)
**KAR4P**			
		SISMA_smile	SISMA_speller
**Box** (3,1)		64 (∼2000)	64 (∼2000)
**Box** (4,1)		256 (∼1000)	256 (∼1000)
**Box** (2,1)		16 (∼6000)	16 (∼6000)
**Structured Motif**			
(3,1) - [2,2]- (4,1) - [2,2] -(3,1) -[1,1] - (2,1)		101,750 (∼ 50)	858 (∼ 50)

The table is divided in three (sub)tables, one for each dataset. The following information apply to each sub-table. There is a row corresponding to each box type involved and one more row corresponding to the type of structured motifs to be found. Also, there is a column for each of the two versions of our SISMA algorithm (SISMA_Smile and SISMA_Speller). Each cell reports two pieces of information: (1) the number of simple/structured motifs in the input sequences that conform to the given specifications, and (2) the corresponding (approximate) average number of occurrences of each simple/structured motif found.

The tools under consideration exhibited very different behaviors on the three datasets, so that there is not a clear “overall” winner.

UASH-URS1-5

SISMA_Smile outperformed Risotto and SISMA_Speller outperformed ExMotif. Running times and differences in running times are really small, meaning that this instance of the problem is not really challenging for any of the tools: SISMA deals with a small number of simple/structured motifs; Risotto drastically reduces the search space starting form the second box on; ExMotif deals with a small number of boxes and allowed substitutions.

UASH-URS1-10

On this dataset SISMA_Speller outperformed ExMotif, while SISMA_Smile without the space-saving option ran out of memory. The reported results refer to SISMA_Smile* with* the option activated (each set $K_{i}$ is partitioned in at least five subsets). SISMA_Smile outperformed Risotto^i^.

This dataset results to be the worst for Risotto, being more affected by the search space size of boxes and the by total number of structured motifs, than by the number of simple motif occurrences.

KAR4P

Risotto and ExMotif outperformed SISMA_Smile and SISMA_Speller, respectively.

SISMA pays a very slow second stage, due to the presence of several thousands of simple motifs occurrences in the input sequences (see Table 2), while Risotto takes advantage from the fact that the occurrence-paths on the suffix tree were significantly less than actual motif occurrences, and search spaces of boxes quite small. Finally, here ExMotif is fast in the phase of neighbor generation, as in this datasets boxes allow at most one error.

**Table 2:** **SISMA**’s running times on biological datasets

	SISMA_Smile		SISMA_Speller	
	1^*st*^ stage	2^*nd*^ stage	1^*st*^ stage	2^*nd*^ stage
**UASH-URS1_5**	0.60 sec	**23.28** sec	**3.00** sec	0.24 sec
**UASH-URS1_10**	0.61 sec	**358.43** sec	5.83 sec	**26.70** sec
**KAR4P**	0.18 sec	**663.04** sec	6.20 sec	**114.64** sec

Running times (in seconds) taken by stage 1 and 2 of SISMA_Smile and SISMA_Speller on biological datasets, when using an uniprocessor machine with 1GB of RAM. With one exception, stage 2 is always (much) slower than stage 1.

## Conclusions

Our conclusion is that the 2-stage approach cannot be turned down without due reflection. In this section we present arguments in support of this thesis and some guidelines that may help the user to choose the most efficient approach, depending on the problem instance s/he has to solve. Finally, we discuss possible improvements of SISMA.

### Direct vs 2-stage approaches

While implementing and working on SISMA we had the opportunity to reason on the advantages and disadvantages of the two approaches, besides running time. 

• *Modularity.* The 2-stage approach is clearly more modular, being made of two (possibly completely) distinct software components. This makes implementation and maintenance easier. Possible optimizations and new variants can be implemented on both stages independently. Stage 1 might be optimized with new, more efficient simple motif extraction tools at negligible costs. The tool might be also easily adapted to extract simple motifs using different algorithms (not only exact), obtaining versions of the tool that tackle slightly different problems. Even more, the tool might be enhanced with the possibility for the user to choose the particular software to run in stage 1.Optimizations and variants might not be equally easy to implement under the direct approach, even though any conclusion to this end strictly depends on the particular software under consideration.

• *Parallelization.* SISMA might be easily adapted to efficiently run on a multiprocessor machine. For stage 1 there may be the availability of a parallel version of the tool adopted (such as PSmile [43]), but otherwise simple motif space enumeration could be easily partitioned and mapped on distinct processors. Stage 2 (lists intersection) might be performed simultaneously on distinct processors as well, by using a technique analogous to the space saving option (with distinct processors accessing to distinct portions of data structures to avoid memory collisions).As before, the design of a fast parallel version of a direct enumeration algorithm cannot be guaranteed without taking the algorithm itself (and its logic) into consideration.

• *Exploratory search of structured motifs.* The 2-stage approach seems to better adapt to an “exploratory search” utilization for structured motif finding (e.g., through a Web interface). The user might be given the possibility to independently execute the two stages (or even upload the simple motifs occurrences); given the results of the first stage, s/he then might run the second stage several times with different input parameters (say, box orders and distance constraints). This feature might be crucial in real applications, where input parameters are difficult to determine with care.This adaptive use of the tool seems really harder to make in case of direct approach without paying a high price in terms of execution times.

• *Search space reduction.* As already pointed out in the paper, since the direct approach looks at the structured motifs as whole, it is able to better handle instances characterized by large size search spaces of some boxes.

### Direct tool comparison

The following observations might be used to guide the user toward the use of one approach/tool or the other. 

• SISMA vs Risotto.

The tests performed show that, when Risotto is faster than SISMA, one or both of the following conditions occur: (1) boxes have small size search spaces and a small number of simple motifs, (2) boxes with large size search spaces occur near the end (large box index) of the structured motifs. Usually, in all the other cases SISMA is faster. This is particularly evident when the structured motifs being found are composed of just one type (or few types) of boxes.Risotto fails for time-out (in our tests, 12 hours), SISMA fails for out-of-memory mainly because of the first stage. Hence, according to the available hardware appropriate decisions on which tool to use can be made.Risotto does not output occurrence positions, but only their number.

• SISMA vs ExMotif.

ExMotif terminates computation only for a small number of boxes and, under this circumstance, it is faster than SISMA only when boxes have small length and/or small number of admitted substitutions. It fails in any other more complex situation, making SISMA_Speller the only available tool for the frequent structured motif discovery problem.

### SISMA implementation and improvements

We designed and developed a tool for exact structured motif discovery, based on the 2-stage approach. Incorporating simple algorithmic ideas and data structures, SISMA is accurately crafted software which proved to compete very well with other published tools for the same problem. On a comprehensive benchmark (composed of both synthetic and real biological datasets) SISMA exhibited more than acceptable performances, even on a very limited power and memory machine. Running times never exceeded the imposed deadline of 12 hours and altogether the tool failed only on few very difficult problem instances (always due to memory shortage).

We can improve SISMA in some respects. As the experiments clearly show, a crucial issue is the possibly high memory consumption during stage 1, which may cause SISMA to fail. The positive side is that memory critical inputs can in general be detected and appropriate actions be taken. One such action consists simply of automatically activating the space saving option. Another option amounts to interleaving simple motif extraction and list intersection. Also, we plan to implement some simple algorithmic improvements that will help to reduce (to some extent) the search space of simple motifs. For instance, we can eliminate proper prefixes of sequences when extracting specific boxes; more precisely, given box *i*, we may cut prefixes that are as long as the sum of the lengths of previous boxes, plus the sum of the minimum distances between previous boxes.

## Endnotes

^a^Search time is sometimes reduced by further constraining the motif definition, as in Weeder [14].

^b^Here we will refer the tool presented in [7] as Speller.

^c^Actually, Ecomp uses an implementation of MITRA-count made by the authors of Ecomp, since MITRA-count itself was, and still is, not available.

^d^Actually, SMILE is a suffix tree-based tool designed to find structured motifs; however it can be used also for simple motif extraction (as a structured motif finder SMILE is outperformed by Risotto).

^e^We discarded the option of implementing a post-processing filter of SMILE output for efficiency reasons, and the option of modifying SMILE code as a more complicated one.

^f^We always have run SISMA with the print option off since Risotto and ExMotif have no possibility to output the starting positions of all occurrences. To be fair, ExMotif actually prints the positions of the exact occurrences.

^g^Observe that direct comparison on average running times might be of little significance, as they might vary considerably on input the same value of *b* (even resulting in charts that might show a non monotonic behavior).

^h^For Risotto failure always means “runs beyond the deadline.”

^i^On machines with more main memory the gap between the running times would have been even more favorable to SISMASmile.

## Competing interests

The authors declare that they have no competing interests.

## Authors’ contributions

All the authors contributed equally to the design of the work. Moreover, PV wrote the software, MF supervised the experiments, ML and MM wrote the paper. All authors read and approved the final manuscript.

## Supplementary Material

Additional file 1 **Implementation details.** Additional file 1 (in pdf format) contains details on the basic implementation of SISMA_Smile and the index box selection variant. Click here for file

Additional file 2 **More experiments on synthetic dataset.** Additional file 2 (in pdf format) includes the results obtained on two more synthetic datasets: one designed as an easier benchmark, one as a particularly hard benchmark for all the tools. Moreover, results are shown for a specific test designed for Risotto, in order to investigate how its performance varies according to boxes order. Click here for file

## References

[B1] Watson JD Baker TA Bell SP Gann A Levine M Losick R Molecular Biology of the Gene6/e: Pearson International Edition; 2007

[B2] Werner T Models for prediction and recognition of eukaryotic promoters Mammalian Genome 1999 10 168 175 10.1007/s003359900963 9922398

[B3] Sinha S Tompa M Discovery of novel transcription factor binding sites by statistical overrepresentation Nucleic Acids Res 2002 30 5549 5560 10.1093/nar/gkf669 12490723 PMC140044

[B4] Lemon B Tjian R Orchestrated response: a symphony of transcription factors for gene control Genes & Dev 2000 14 2551 2569 10.1101/gad.831000 11040209

[B5] Wray GA The evolutionary significance of cis-regulatory mutations Nature Rev Genet 2007 8 206 216 17304246 10.1038/nrg2063

[B6] Bailey TL Elkan C The Value of Prior Knowledge in Discovering Motifs with MEME Proceedings of 3rd International Conference on Intelligent Systems for Molecular Biology (ISMB ’95) 1995 21 29 7584439

[B7] Sagot MF Spelling approximate repeated or common motifs using a suffix tree Lecture Notes Comput Sci 1998 1380 111 127

[B8] Li M Ma B Wang L Finding Similar Regions in Many Strings Proceedings of the 31th Annual ACM Symposium on Theory of Computing (STOC ’99) 1999 473 482

[B9] Lawrence CE Altschul SF Boguski MS Liu JS Neuwald AF Wootton JC Detecting subtle sequence signals: a Gibbs sampling strategy for multiple alignment Science 1993 262 208 214 10.1126/science.8211139 8211139

[B10] Brazma A Jonassen I Eidhammer I Gilbert D Approaches to the Automatic Discovery of Patterns in Biosequences J Comput Biol 1998 5 2 277 304 http://citeseer.ist.psu.edu/article/brazma97approaches.html 10.1089/cmb.1998.5.279 9672833

[B11] van Helden J André B Collado-Vides J Extracting regulatory sites from the upstream region of yeast genes by computational analysis of oligonucleotide frequencies Mol Biol 1998 281 827 842 http://citeseer.ist.psu.edu/biol02extracting.html 10.1006/jmbi.1998.1947 9719638

[B12] Pevzner PA Sze SH Combinatorial Approaches to Finding Subtle Signals in DNA Sequences Proceedings of 8th International Conference on Intelligent Systems for Molecular Biology (ISMB ’00) 2000 269 278 10977088

[B13] Guha-Thakurta D Stormo GD Identifying target sites for cooperatively binding factors Bioinformatics 2001 17 608 621 10.1093/bioinformatics/17.7.608 11448879

[B14] Pavesi G Mauri G Pesole G An algorithm for finding signals of unknown length in DNA sequences Bioinformatics 2001 17 207 214 10.1093/bioinformatics/17.suppl_1.S207 11473011

[B15] Eskin E Pevzner P Finding composite regulatory patterns in DNA sequences Proceedings of the 10th Annual International Conference on Intelligent Systems for Molecular Biology (ISMB ’02) 2002 S354 S363 10.1093/bioinformatics/18.suppl_1.s354 12169566

[B16] Sinha S Tompa M YMF: a program for discovery of novel transcription factor binding sites by statistical overrepresentation Nucleic Acids Res 2003 31 3586 3588 10.1093/nar/gkg618 12824371 PMC169024

[B17] Leung HCM Chin FYL Generalized Planted (l, d)-Motif Problem with Negative Set Proceedings of the Workshop on Algorithms in Bioinformatics (WABI) 2005 264 275

[B18] Favorov AV Gelfand MS Gerasimova AV Ravcheev DA Mironov AA Makeev VJ A Gibbs sampler for identification of symmetrically structured, spaced DNA motifs with improved estimation of the signal length Bioinformatics 2005 21 2240 2245 10.1093/bioinformatics/bti336 15728117

[B19] Mendes N Casimiro A Santos P Sá-Correia I Oliveira A Freitas A MUSA: a parameter free algorithm for the identification of biologically significant motifs Bioinformatics 2006 22 2996 3002 10.1093/bioinformatics/btl537 17068086

[B20] D’haeseleer P How does DNA sequence motif discovery work? Nat Biotech 2006 24 8 959 961 http://dx.doi.org/10.1038/nbt0806-959 10.1038/nbt0806-959 16900144

[B21] Das MK Dai HK A survey of dna motif finding algorithms BMC Bioinformatics 2007 8 S21 18047721 10.1186/1471-2105-8-S7-S21 PMC2099490

[B22] Stormo GD Hartzell GW III Identifying protein binding sites from unaligned DNA fragments PNAS 1989 86 1183 1187 10.1073/pnas.86.4.1183 2919167 PMC286650

[B23] Wolfertstetter F Frech K Herrmann G Werner T Identification of functional elements in unaligned nucleic acid sequences by a novel tuple search algorithm Comput Appl Biosci 1996 12 71 80 8670622 10.1093/bioinformatics/12.1.71

[B24] Tompa M An exact method for finding short motifs in sequences, with application to the ribosome binding site problem Proceedings of 7th International Conference on Intelligent Systems for Molecular Biology (ISMB ’99) 1999 262 271 10786309

[B25] Linhart C Halperin Y Shamir R Transcription factor and microRNA motif discovery: The Amadeus platform and a compendium of metazoan target sets Genome Res 2008 18 7 1180 1189 10.1101/gr.076117.108 18411406 PMC2493407

[B26] Zhang Y Zaki MJ EXMOTIF: efficient structured motif extraction Algorithms Mol Biol 2006 1 21 10.1186/1748-7188-1-21 17109757 PMC1698483

[B27] Pisanti N Carvalho A Marsan L Sagot MF RISOTTO: Fast extraction of motifs with mismatches Proceedings of the 7th Latin American Theoretical Informatics Symposium 2006

[B28] Zhou J Sander J Lin G Efficient composite pattern finding from monad patterns Int J Bioinf Res Appl 2007 3 86 99 10.1504/IJBRA.2007.011836 18048174

[B29] Tompa M Li N Bailey TL Church GM et. al Assessingcomputational tools for the discovery of transcription factor binding sites Nature Biotechnol 2005 23 137 144 http://www.ncbi.nlm.nih.gov/pubmed/15637633 10.1038/nbt1053 15637633

[B30] McCreight EM A Space-Economical Suffix Tree Construction Algorithm J ACM 1976 23 2 262 272 10.1145/321941.321946

[B31] Gusfield D Algorithms on Strings, Trees, and Sequences: Computer Science and Computational Biology 1997 New York: Cambridge University Press

[B32] Marsan L Sagot MF Algorithms for Extracting Structured Motifs Using a Suffix Tree with an Application to Promoter and Regulatory Site Consensus Identification J Comput Biol 2000 7 3-4 345 362 10.1089/106652700750050826 11108467

[B33] Carvalho A Freitas A Oliveira A Sagot MF A highly scalable algorithm for the extraction of cis-regulatory regions Proceedings of the Asia-Pacific Bioinformatics Conference 2005 273 282

[B34] Allali J Sagot MF The at most k-deep factor tree. Tech 2004

[B35] Carvalho A Freitas A Oliveira A Sagot Efficient Extraction of Structured Motifs Using Box-links Proceedings of 11th Conference on String Processing and Information Retrieval 2004 267 268 http://citeseer.ist.psu.edu/viewdoc/summary?doi:10.1.1.102.9439

[B36] Leung CM Chin FYL Algorithms for Challenging Motif Problems J Bioinf Comput Biol 2006 4 43 58 10.1142/S0219720006001692 16568541

[B37] Buhler J Tompa M Finding motifs using random projections J Comput Biol 2002 9 225 242 10.1089/10665270252935430 12015879

[B38] Davila J Balla S Rajasekaran S Fast and Practical Algorithms for Planted (l,d)-Motif Search IEEE/ACM Trans Comput Biol Bioinf (TCBB) 2007 4 4 544 552 10.1109/TCBB.2007.70241 17975266

[B39] Federico M Valente P Leoncini M Montangero M Cavicchioli R An Efficient Algorithm for Planted Structured Motif Extraction CompBio ’09: Proceedings of the 1st ACM Workshop on Breaking Frontiers of Computational Biology 2009 1 6

[B40] Zhu J Zhang M SCPD: a promoter database of the yeast Saccharomyces cerevisiae Bioinformatics 1999 15 607 611 10.1093/bioinformatics/15.7.607 10487868

[B41] Teixeira MC Monteiro P Jain P Tenreiro S Fernandes AR Mira NP Alenquer M Freitas AT Oliveira AL Sá-Correia I The YEASTRACT database: a tool for the analysis of transcription regulatory associations in Saccharomyces cerevisiae Nucleic Acids Res 2006 34 D446 D451 10.1093/nar/gkj013 16381908 PMC1347376

[B42] Thomas-Chollier M Sand O Turatsinze JV Janky R Defrance M Vervisch E Brohee S van Helden RSAT: regulatory sequence analysis tools Nucleic Acids Res 2008 36 W119 W127 10.1093/nar/gkn304 18495751 PMC2447775

[B43] Carvalho AM Freitas AT Oliveira AL Sagot MF A parallel algorithm for the extraction of structured motifs Proceedings of the 19th ACM Symposium on Applied Computing (SAC’04) 2004 147 153

