# Supplementary material for: Direct vs 2-stage approaches to structured motif finding
Source: Algorithms Mol Biol. 2012 Aug 21;7:20. doi: 10.1186/1748-7188-7-20 (PMC3564690; doi:10.1186/1748-7188-7-20)
Supplement: Additional file 1 — Implementation details. Additional file 1 (in pdf format) contains details on the basic implementation of SISMA_Smile and the index box selection variant. [file 1748-7188-7-20-S1.pdf]

Additional File 1

Direct vs 2-Stage Approaches to Structured Motif Finding

Maria Federico, Mauro Leoncini, Manuela Montangero, and Paolo Valente

SISMA implementation details

Basic Implementation

Figure 1 shows the vector data structures that are used to store information on (A) simple and (B) structured motifs.

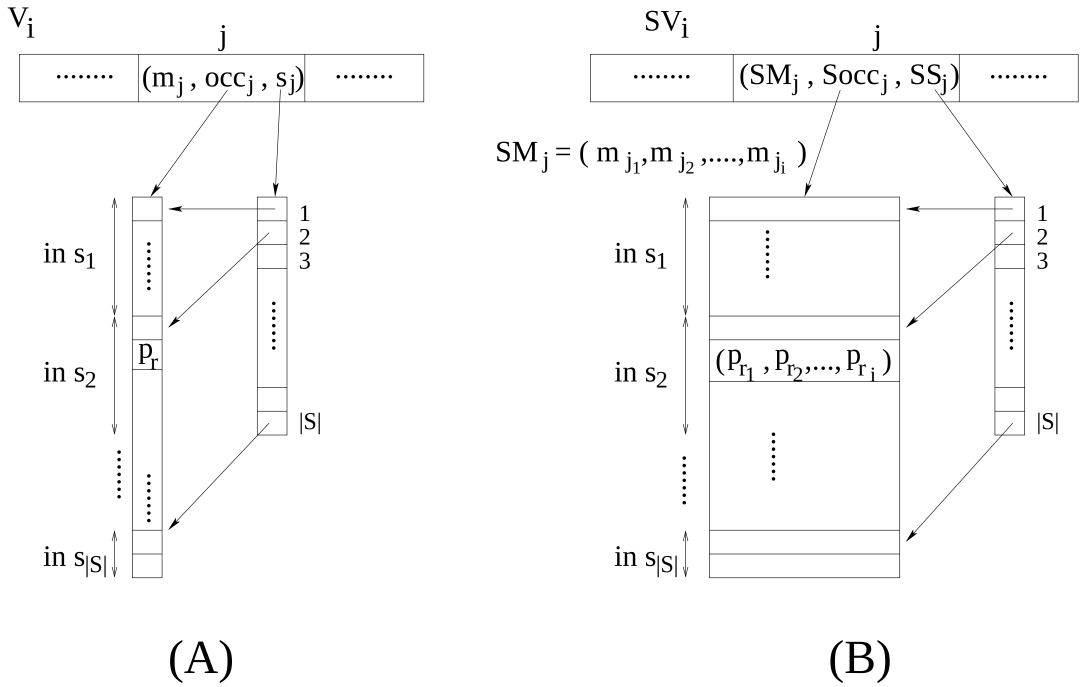

Figure 1: Data structures used by SISMA to store information concerning (A) simple and (B) structured motifs.

Data structure for simple motifs. Given an instance of the structured motif discovery problem, let K_i denote (as in the paper) the set of simple motifs that are solutions to the simple motif discovery problem with parameters S (set of sequences), q (quorum), ℓ_i and e_i (motif length and number of admitted errors), for any $i \in \{1, \ldots, b\}$. We represent each K_i using a vector V_i that stores the triplets (m_j, occ_j, S_j), for $j = 1, \ldots, |K_i|$, such that: $m_j \in K_i$ is a simple motif; occ_j is a vector storing the positions of all occurrences of m_j in S, ordered by increasing input sequence index and by increasing position in each sequence; S_j is a vector, indexed by sequence indices, such that $S_j[z]$ is the index, in occ_j, of the first occurrence of m_j appearing in sequence s_z, for $z = 1, \ldots, |S|$; $S_j[z] =$NULL if there are no occurrences of the structured motif in sequence s_z.

Data structure for structured motifs. Structured motifs composed of i boxes ($i > 1$), together with all their occurrences, are stored in the vector data structure SV_i in the following way: the cell at generic index j of the vector stores a triplet $(SM_j, Socc_j, SS_j)$, with the meaning defined below.

1. $SM_j = (m_{j_1}, \ldots, m_{j_i})$ is a structured motif composed by i boxes. In particular, given box index $t \in [1, i]$, we have that j_t is the index of a simple motif in the set K_t that might occur in the box indexed by t.

2. $Socc_j$ is a vector of pairs storing the positions of the first box and of the last box of the occurrences of SM_j on a specific sequence. When the *box index selection* option is activated, $Socc_j$ stores the starting positions of all boxes analyzed so far.

3. SS_j is a vector, indexed by sequence indices, such that $SS_j[z]$ is the index in $Socc_j$ of the first occurrence of tuple SM_j appearing in sequence s_z; $SS_j[z] =$NULL if there are no occurrences of the structured motif in sequence s_z.

List intersection performed at generic step i of the second stage. Given an index r, $SV_i[r]$ stores the i-tuple of simple motifs $SM_j = (m_{r_1}, \ldots, m_{r_i})$ representing a structured motif with i boxes that SISMA has already computed in the previous step, together with all its occurrences; given index v, $V_{i+1}[v]$ stores the simple motif $m_v \in K_{i+1}$, together with all its occurrence positions into vector occ_v. SISMA performs the two following operations:

- *Distance constraint check.* For each sequence $s_t \in S$, SISMA looks for the first occurrence of m_v that satisfies the distance constraint. This is done efficiently by performing a binary search on the portion of vector occ_v relative to sequence s_t (*i.e.*, going from index $S_v[t]$ to index $S_v[t+1]-1$), to find the first occurrence starting position greater or equal to $p_i + |m_{r_i}| + d_{min_i}$. If found, for the successive occurrences of the simple motif, SISMA just checks if their starting position is smaller or equal to $p_i + |m_{r_i}| + d_{Max_i}$. As soon as this last condition is violated, SISMA proceeds to examine the occurrences (both of the structured and simple motifs) on the subsequent sequence.

- *Quorum constraint check.* At each step, SISMA keeps only the structured motifs that satisfy the quorum constraint; *i.e.*, those for which the vector SS_j contains at least q non NULL pointers.

Box index selection option

When the box index selection option is activated, in the second stage, SISMA selects boxes for list intersection by increasing total number of occurrences, and not by increasing index order; *i.e.*, for any index $z \in [1..b]$ SISMA computes the following quantity:

$$occM_z = \sum_{t=0}^{|V_z|-1} |occ_{z,t}|,$$

that is the total number of occurrences stored in each vector V_z. Indices are, then, ordered according to increasing $occM$s and selected according to this order in the successive steps of stage 2.

Step i of stage two ($i = 1, \ldots, b-1$): we are given a set of indices $B_i \subseteq [1..b]$, such that $|B_i| = i$, corresponding to boxes that have already been taken into consideration in previous steps. Notice that indices in B_i do not need to be consecutive indices. In the first step (step 1), we have that $B_1 = \{\hat{\imath}\}$, for some $\hat{\imath} \in [1..b]$, that is the first index according to the $occM$s order.

First SISMA selects the next box index $\hat{\jmath} \in [1..b] \setminus B_i$, according to the $occM$s order. Then, for any structured motif computed at the previous step, and for any simple motif stored in $V_{\hat{\jmath}}$, SISMA tries to compose new structured motifs of $i+1$ boxes, by checking distance and quorum constraints on the combination of all occurrences of the simple and the structured motifs.

Structured motifs composed of i boxes, and all their occurrences, are stored in the vector data structure SV_i very similar to the one used in the basic implementation (see Figure 1.B). The vector stores triplets $(SM_j, Socc_j, SS_j)$ in which the difference with the basic implementation concerns the tuple SM_j that contains motifs that are placed in boxes that do not necessarily have consecutive box indices. This reflects in the fact that vector of occurrence positions $Socc_j$ stores i-tuples (and not only pairs) containing the starting positions of the simple motif of each box of the structured motif.

To perform list occurrence, SISMA takes into consideration all pairs composed by one structured motif computed in the previous step and one simple motifs selected by index $\hat{\jmath}$. For each such pair, SISMA considers all pairs of occurrences of structured and simple motifs that appear on the same sequence and checks if it is possible to build an occurrence, on that sequence, of a new structured motif with $i+1$ boxes.

In particular, given index r, $SV_i[r]$ stores the i-tuple of simple motifs $SM_r = (m_{r_1}, \ldots, m_{r_i})$ representing a structured motif with i boxes that SISMA has already computed in step i, together

with all its occurrences. We have that $r_j \in B_i$ for all $j \in [1,i]$ and that $r_{j-1} < r_j$ for all $j \in [2,i]$.[1] Given index v, $V_{\hat{j}}[v]$ stores the simple motif $m_v \in K_{\hat{j}}$, together with all its occurrences into vector occ_v.

Let r_{pred} be the largest box index in B_i such that $r_{pred} < \hat{j}$ and let r_{succ} be the smallest box index in B_i such that $\hat{j} < r_{succ}$. Observe that at least one such index surely exists, w.l.o.g assume at first that both indices exist, and that the three indices $r_{pred}, \hat{j}$ and r_{succ} might not be consecutive.

SISMA tries to build an $(i+1)$-boxes structured motif in which simple motifs appear in the following order:

$$(m_{r_1}, \ldots, m_{r_{pred}}, m_v, m_{r_{succ}}, \ldots, m_{r_i}).$$

The implementation of this option introduces significant changes in the distance constraint check procedure, while the rest of the successive operations requires only minor changes.

Distance constraint check. For each occurrence of the structured motif SM_r, SISMA checks which occurrence of the simple motif satisfies distance constraints; *i.e.*, if it is in between the current occurrences of $m_{r_{pred}}$ and $m_{r_{succ}}$, according to constraints given by pairs(d_{min}, d_{Max})s.

Formally, given index g, then $Socc_r[g] = (p_{r_1}, \ldots, p_{r_i})$ stores the starting positions of one occurrence of the structured motif SM_r (of simple motifs of that particular occurrence) on a particular sequence s_z; *i.e.*, p_{r_k} is the starting position of m_{r_k} in s_z.

Let f be the index in occ_v of the starting position p_v of one particular occurrence of the simple motif $m_v \in K_{\hat{j}}$ on the same sequence s_z; *i.e.*, it holds that $occ_v[f] = p_v$.
There is an occurrence of the structured motif with $i+1$ boxes if distance constraints are not violated; *i.e.*, if the two following conditions hold (see Figure 2 for a geometric interpretation):

- the occurrence of m_v starting at p_v is not too close and not too far away from the occurrence of $m_{r_{pred}}$:

$$\underbrace{p_{r_{pred}} + \sum_{w=pred}^{\hat{j}-1} (|m_w| + d_{min_w})}_{(a)} \leq p_v \leq \underbrace{p_{r_{pred}} + \sum_{w=pred}^{\hat{j}-1} (|m_w| + d_{Max_w})}_{(b)} \quad (1)$$

- the occurrence of m_v starting at p_v is not too far away and not too close from the occurrence of $m_{r_{succ}}$:

[1]For the sake of presentation we omitted a double indices in the notation of SM_r. A more correct formulation would have been to denote SM_j as an i-tuple $(m_{t_1,j_1}, \ldots, m_{t_i,j_i})$ of simple motifs extracted in stage 1 of SISMA, whose corresponding boxes have already been taken into consideration in the previous steps of stage 2. For any $r = 1, \ldots, i$, given a box index $t_r \in B_i$, we have that the simple motif $m_{t_r,j_r} \in K_{t_r}$ occurs in the box that is in position t_r of the structured motif. Simple motifs in the tuple $SM_{i,j}$ are stored by increasing box index (*i.e.*, $t_r < t_{r+1}$ for $r = 1, \ldots, i-1$).

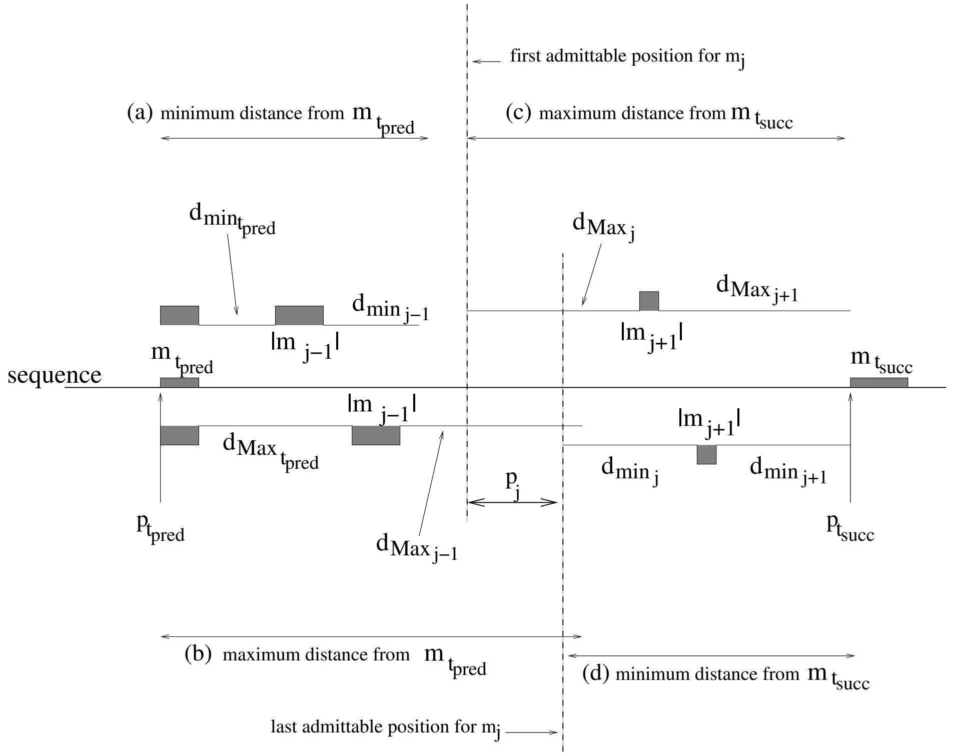

Figure 2: Geometric interpretation of equations (1) and (2). In this example we have $r_{pred} = \hat{\jmath} - 2$ and $r_{succ} = \hat{\jmath} + 2$. The position of $m_{r_{pred}}$ and $m_{r_{succ}}$ is depicted on the sequence. Above the sequence: (a) the minimum distance that m_v must have from $m_{r_{pred}}$; (c) the maximum distance that m_v might have from $m_{r_{succ}}$. Under the sequence: (b) the maximum distance that m_v might have from $m_{r_{pred}}$; (d) the minimum distance that m_v must have from $m_{r_{succ}}$. The segment on the sequence that is in between the dashed lines is the region where to check for an occurrence of m_v.

$$\underbrace{p_{r_{succ}} - \sum_{w=\hat{\jmath}}^{succ-1} \left(d_{Max_w} + |m_w|\right)}_{(c)} \leq p_v \leq \underbrace{p_{r_{succ}} - \sum_{w=\hat{\jmath}}^{succ-1} \left(d_{min_w} + |m_w|\right)}_{(d)}. \tag{2}$$

If index r_{pred} (or r_{succ}) does not exist, only the right side (or left side) of the equations must hold.

As for the basic implementation, also in this case, to reduce the number of useless checks and make the procedure more efficient, SISMA implements the former checks by looking for the first occurrence of m_v that might satisfy distance constraint by performing a binary search on the portion

of vector occ_v concerning sequence s_z; *i.e.*, form index $S_v[z]$ to index $S_v[z+1]-1$. Remember that occurrences in occ_v are stored, sequence by sequence, by increasing position order.

In particular, SISMA looks for the first index f in occ_v that satisfies the left part of equations (1) and (2), that is, index f such that

$$occ_v[f] \geq \max\{(a),(c)\}\,. \tag{3}$$

Once index f has been found, the distance constraint check starts with the occurrence stored at index f and continues with occurrences at successive positions, until position $S_v[z+1]$ is reached (end of sequence) or distance constraint (3) is violated. Observe that, once f has been found, it is sufficient to check that the right side of equations (1) and (2) hold, *i.e.*, that, given index f' such that $f \leq f' \leq S_v[z+1]$ we have

$$occ_v[f'] \leq \min\{(b),(d)\}\,.$$

Finally, observe that summations (and summations only) in equations (1) and (2) have to be computed only once at each step, as they depend on box index $\hat{\jmath}$ and not on particular occurrences of particular motifs. On the contrary, $p_{r_{pred}}$ and $p_{r_{succ}}$ strongly depend on the particular occurrence of the structured motif we are taking into consideration.
