# Supplementary material for: Direct vs 2-stage approaches to structured motif finding
Source: Algorithms Mol Biol. 2012 Aug 21;7:20. doi: 10.1186/1748-7188-7-20 (PMC3564690; doi:10.1186/1748-7188-7-20)
Supplement: Additional file 2 — More experiments on synthetic dataset. Additional file 2 (in pdf format) includes the results obtained on two more synthetic datasets: one designed as an easier benchmark, one as a particularly hard benchmark for all the tools. Moreover, results are shown for a specific test designed for Risotto, in order to investigate how its performance varies according to boxes order. [file 1748-7188-7-20-S2.pdf]

Additional File 2

Direct vs 2-Stage Approaches to Structured Motif Finding

Maria Federico, Mauro Leoncini, Manuela Montangero, and Paolo Valente

More experiments on synthetic dataset

In this additional file we report results for extra experiments that we made on synthetic data. In particular:

- We tested the tools on possibly easy instance, where strucutred motifs are composed by a different number of boxes but are all of the same type (*i.e.*, same lenght and number of errors).
- We stressed tools with possibly very hard instances, according to the PMP classification, in which the number of allowed substitutions was randomly chosen as a function of box length.
- We tested the influence of difficult boxes, according to their position in the planted structured motif, on the tools' performance.

Planted structured motifs with boxes having same length and errors

We generated datasets using, for each dataset, a fixed template (ℓ, e) for each box. The value of ℓ varied from 9 to 15 in different experiments, with $e \in \{1..\lfloor 1/3\ell \rfloor\}$, and with the number of boxes ranging from 2 to 10.

For each choice of the parameters we performed 20 runs for each tool, computing the average running time and the corresponding 95% confidence interval.

As a general conclusion we may notice that, under these experimental conditions, SISMA tools outperformed the competitors in essentially all datasets on which tools ended comutation within the imposed deadline. Figure 1 reports the results obtained for the pairs $(10, 3)$, $(11, 3)$, $(12, 3)$, $(13, 3)$, and $(15, 5)$ (vertical bars represent the 95% confidence interval of the average running times). Such pairs are typical of the observed behaviors of the four tools within this experimental setting. For what concerns the direct tool comparison, we can make the following observations.

SISMA_Smile vs Risotto. SISMA_Smile always outperforms Risotto when both tools end computation. On few instances, however, while Risotto does not end within the deadline, SISMA_Smile fails because of memory shortage (even with the space-saving option selected). On the other hand, it never happens that SISMA_Smile fails and Risotto ends within the imposed deadline (12 hours).

The prevalence of SISMA_Smile over Risotto on these datasets mainly depends on the fact that SISMA_Smile explores the search space of simple motifs just once (with a single call to smile). On the contrary, Risotto explores the search space for each box, even though this becomes narrower as boxes are added.

The situation gets even worse for Risotto when the search space to be explored is large, as for instances characterized by a large number of expected motifs. In particular, Risotto goes in time-out already with two boxes in the following cases: $(9, 3)$,$(10, 3)$,$(12, 4)$, $(15, 4)$ and $(15, 5)$. This is coherent with the fact that Risotto's running time is exponential with box length and with the number of errors.

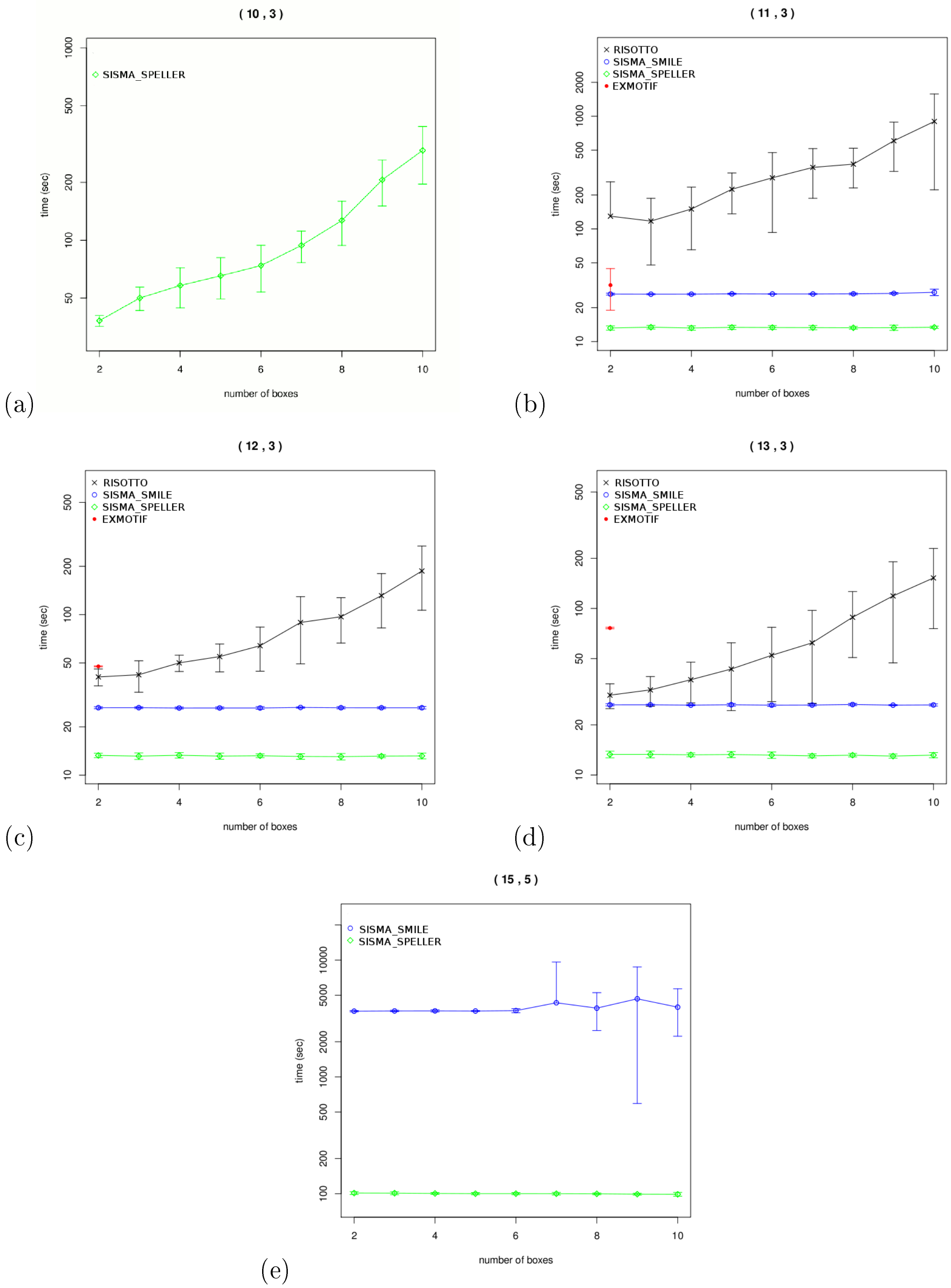

Figure 1: Average running times (in seconds) for the four tools on instances generated using pairs (a) $(10, 3)$, (b) $(11, 3)$, (c) $(12, 3)$, (d) $(13, 3)$ and (e) $(15, 5)$. Vertical bars represent the 95% confidence interval. When tools do not end computation, results are not reported.

SISMA_Smile goes out of memory, already for two boxes, only for instances characterized by pairs $(9,3),(10,3)$ and $(12,4)$, even in case of the space-saving implementation.

SISMA_Speller vs ExMotif. In Table 1 we give a wide summary of the comparison between these tools, reporting whether tools terminate and which tool is the fastest.

ℓ	9 -13	14	15
b, e	$e = 1$<br>$b \leq 3$ ExMotif always faster<br>$b = 4$ essentially equal<br>$b \geq 5$ SISMA always faster<br>$b \geq 6$ ExMotif always fails<br>$e = 2$<br>$b = 2$ ExMotif always faster<br>$b \geq 3$ SISMA always faster<br>$b \geq 4$ ExMotif always fails<br>$e \geq 3$<br>SISMA always faster, ExMotif ends only for $b = 2$<br>SISMA fails only for $(9,3)$	SISMA always faster<br>ExMotif fails for<br>$e = 1$ and $b \geq 5$<br>$e = 2$ and $b \geq 3$<br>$e \geq 3$	SISMA/ExMotif always faster/fails

As it can be easily seen, in the majority of runs SISMA_Speller outperforms ExMotif. Exceptions occur only in few particular cases, with small number of boxes and few substitutions allowed. ExMotif turns significantly slower than SISMA_Speller when the structured motifs have three boxes or more, and in general when any of the input parameters (number of boxes, box length, and number of allowable substitutions) increases.

SISMA_Speller always ends its computation, with the exception of the instances characterized by the pair $(9,3)$. Its running time is generally below one minute for "easy" instances, never above five minutes for "hard" instances (e.g., 10 boxes characterized by length 10 and 3 allowed errors). In case of $(9,3)$ simple motifs, SISMA_Speller fails even in case of two boxes and even if the output is produced in slices *i.e.*, with the space-saving option on. Such failure depends on the high number (more that 10^4) of simple motifs, which makes the program go out of memory when looking for dyads.

Planted structured motifd with boxes having different leghts and errors

To stress tools with particularly difficult instances, we ran another set of synthetic experiments in which we randomly planted simple motifs in the sequences with length ℓ varying from 7 to 18, and errors in the range $[1..\lfloor \ell/3 \rfloor]$. SISMA_Smile was run with the box index selection option activated.

As for the experiments presented in the regular paper, we compared tools' performance using running times and counting how many times one tool outperformed the other.

Risotto vs SISMA_Smile. Figure 2 shows (a) the number of times in which one tool outperformed the other, and (b) the number of failures for each tool. Again, failures might not sum up to the total number of runs.

In this experimental setting, running times varied considerably from instance to instance, even for the same number of boxes. With respect to experiments shown in the paper (Section *Test on synthetic data*), here this is much more evident.

We observe that we have a larger number of failures, with respect to the other set of synthetic experiments with variable boxes. This was to be expected since some instances here are much more challenging. In particular, we notice that Risotto starts failing already from three boxes, while SISMA_Smile exhibits a slightly better behavior (with the exception of five boxes where we observe many failures for both tools, meaning that several hard instances were randomly selected).

Moreover, SISMA_Smile fails for memory shortage only, and always because of the first stage (i.e., too many simple motifs were found and they could not fit into primary memory). This kind of failures usually happens much earlier than the established deadline of 12 hours.

On the other hand, Risotto fails always due to time-out, hence 12 hours were actually a lower bound on running time for those instances. There are two main reasons why Risotto fails: (1) in one of the first positions of the structured motif there is box with a large search space (but not necessarily a large number of motifs); (2) the output is very large.

In the first case, if the actual number of simple motifs found is not large, SISMA_Smile is usually slow, but nonetheless it ends computation before time-out. In the latter case, the second stage of SISMA_Smile is usually long, but neither in this cases more than 12 hours. As previously pointed out, the worst case for SISMA_Smile usually happens when the number of simple motifs found in the first stage is large, but not enough to cause out-of-memory.

The typical case in which Risotto outperforms SISMA_Smile is when there is a box, in the last positions of the structured motifs, with large search space and a small number of simple motifs. Risotto drastically reduces the search space, while the running time of SISMA_Smile is almost entirely dedicated to the extraction of simple motifs for such boxes.

Figure 3 and Figure 4 show best, worst, average running times and standard deviation when considering all runs and when considering only runs in which both tools end computation. In the first case, 12 hours are counted as a lower bound for Risotto running time, when this fails due to time-out; when SISMA_Smile fails for out-of-memory, we considered the actual run time (never exceeding 12 hours). Again, as Risotto is the tool failing the most, the differences in the charts are more evident for Risotto than for SISMA_Smile.

Observe that, in Figure 3.a and Figure 4.a', for ten boxes, the best case for Risotto changes (it is worst in the second figure), meaning that the best case for Risotto is one in which SISMA fails. Analogously, for worst case, Figure 3.b and Figure 4.b', there is a differences for SISMA for five and six boxes. In these cases we have shorter runs, meaning that the instances on which Risotto failed are somehow difficult also for SISMA (that, nevertheless, ended computation before 12 hours).

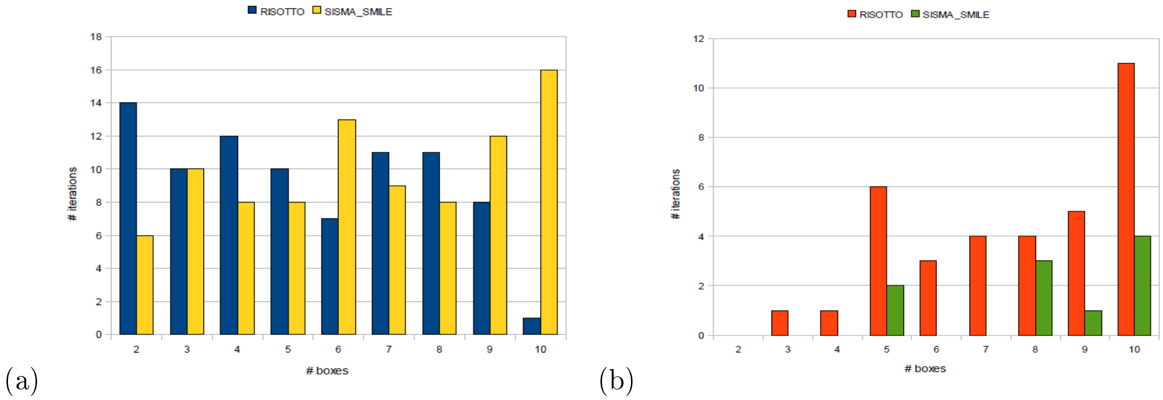

Figure 2: (a) Number of times in which one tool outperformed the other. (b) Number of tool failures.

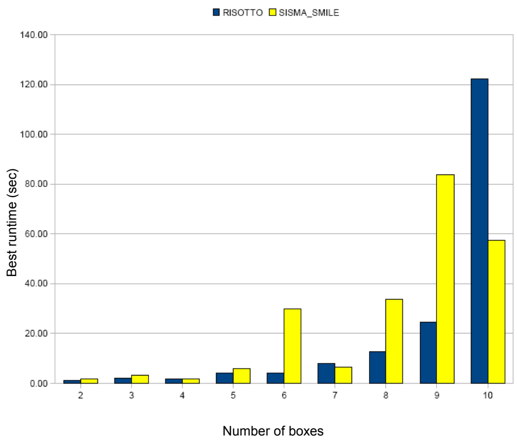

(a)

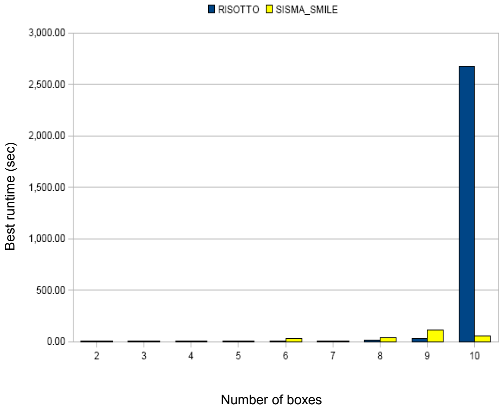

(a’)

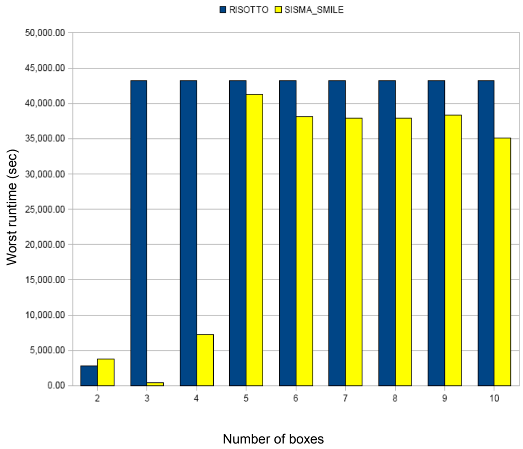

(b)

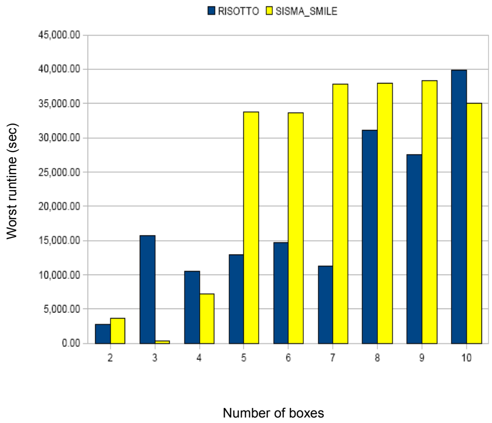

(b’)

Figure 3: (Best and Worst runtimes (in seconds) for SISMA_SMILE and RISOTTO, when considering (a), (b) all runs and (a’), (b’) only runs in which both tools ended computation.

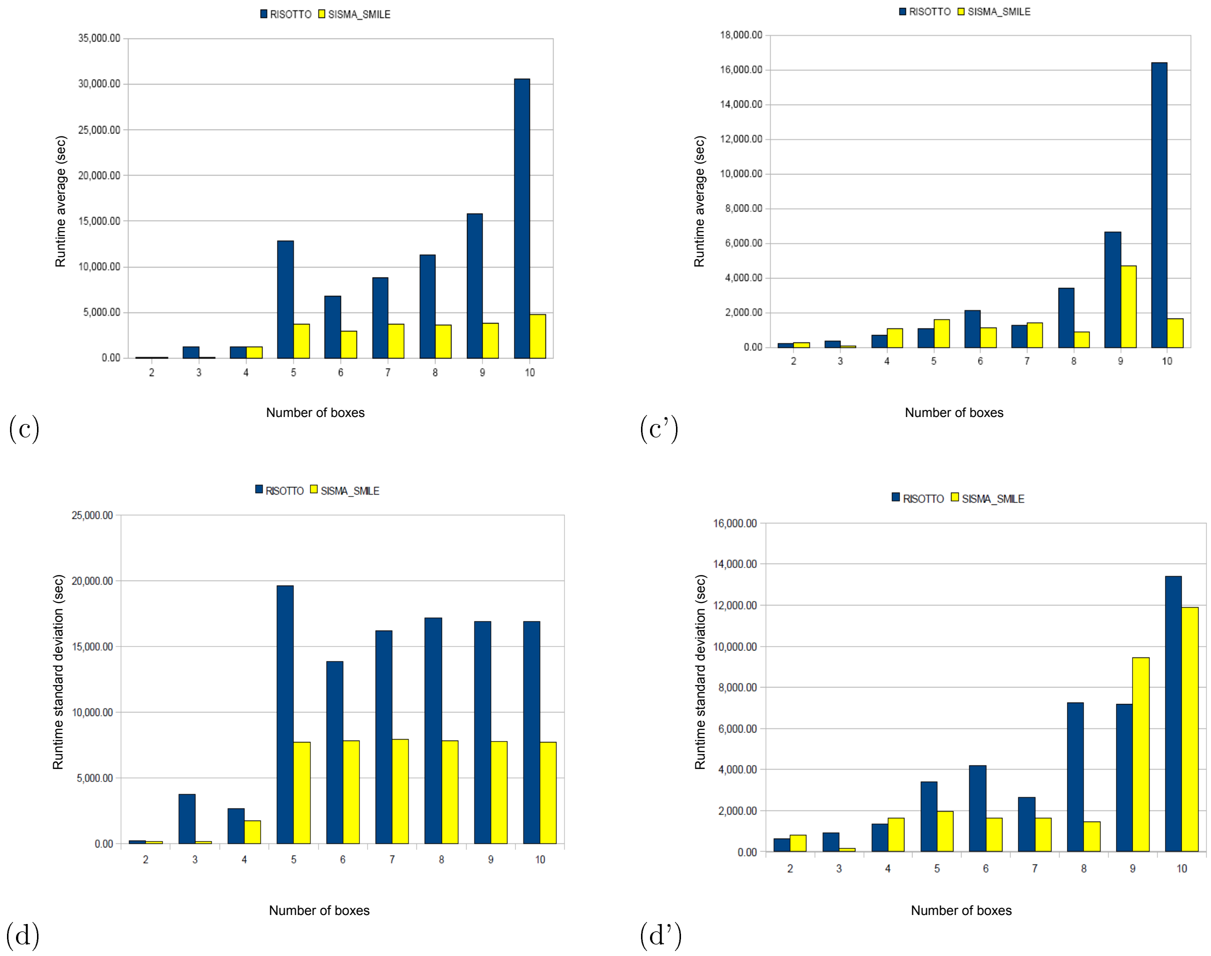

Figure 4: Average runtimes and standard deviation (in seconds) for SISMA_Smile and Risotto when considering (c), (d) all runs and (c'), (d') only runs in which both tools ended computation. Average runtimes and standard deviations have been computed omitting best and worst run.

As in this set of experiments we have a larger number of failures, we also investigated the behavior of the tool that did not fail when the other did. Figure 5 shows best, worst, average running times (in seconds) and standard deviation of Risotto and SISMA on the runs in which the other tool failed. When only the best running time is reported, we have only one run. As we have a small number of runs to analyze, averages and variances have been computed considering all runs.

ExMotif vs SISMA_Speller. Differently of the other set of experiments, here ExMotif does end computation in few cases (namely 12 over 200 tests, 6% of the times), but never for tests with number of boxes $b \geq 4$. Among those tests in which it ends, three times its running time is shorter than SISMA_Speller running time. We are, nevertheless, talking about runs never exceeding 48 seconds.

Figure 6 reports best, worst, average running times (in seconds) and standard deviation of SISMA_Speller in this set of experiments. We can see that running time is always extremely competitive, the worst run (for five boxes, due to the presence of box $(17, 5)$, having extremely large

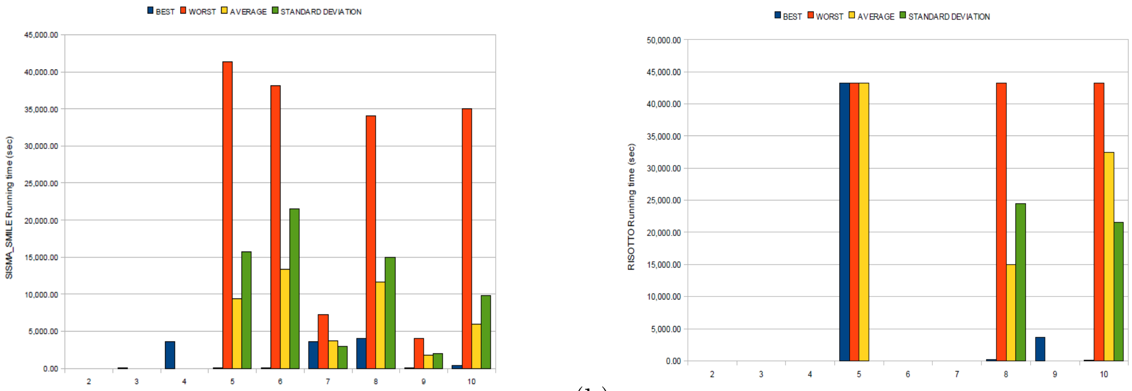

(a) (b)

Figure 5: Best, Worst, Average runtimes and standard deviation for (a) SISMA_Smile and (b) Risotto when the other tool fails. If only best running time is shown, there is only one run for that number of boxes. Means and standard deviations have been computed using all values.

Box (14,4) position	1	2	3	4	5	6	7	8	9
SISMA 1st stage	394.45	378.54	379.92	376.98	378.30	379.63	377.69	375.60	378.87
SISMA 2st stage	1.28	1.28	0.42	0.20	0.24	0.24	0.22	0.12	0.08
SISMA total time	**395.73**	**379.82**	380.34	377.18	378.54	379.87	377.91	375.72	378.95
Risotto time	790.15	2957.36	**135.49**	**114.68**	**98.66**	**77.80**	**58.10**	**60.46**	**59.60**

Table 1: Running times (in seconds) of experiments performed on nine different synthetic datasets containing 20 random sequences of length 600 in which nine boxes characterized by pairs $(14,4)$, $(12,3)$, $(12,2)$, $(10,2)$, $(10,2)$, $(10,2)$, $(9,2)$, $(10,2)$, $(13,2)$ and generated moving the $(14,4)$ box at different positions in the structured motif, from 1 to 9, are implanted at random positions into sequences. When the $(14,4)$ is in the first or second position SISMA_Smile outperforms Risotto.

search space) takes 12 min and 24 sec, on average runs take around 100 sec for number of boxes form five on.

About boxes positions in structured motifs

A closer inspection on Risotto's behavior shows that its running time may be highly affected by the positions of boxes with large search space, such as $(14,4)$. Table 2 shows the results of a set of experiments performed on nine different synthetic datasets. Each dataset contained 20 random sequences of length 600 in which we planted a structured motif composed of the following nine boxes: $(14,4)$, $(12,3)$, $(12,2)$, $(10,2)$, $(10,2)$, $(10,2)$, $(9,2)$, $(10,2)$, $(13,2)$. The relative positions of all the boxes, except $(14,4)$, was the same in all datasets, while the $(14,4)$ box moved from position 1 to 9.

As we can see, all the datasets cause SISMA_Smile to spend the vast majority of the running time in stage 1. The latter lasts almost the same time independently of the box order and altogether SISMA_Smile running time varies only 20 sec from best to worst run[1]. On the contrary, the

[1]The differences depends on the fact that the planted motifs for a given box (ℓ, e) were possibly not the same.

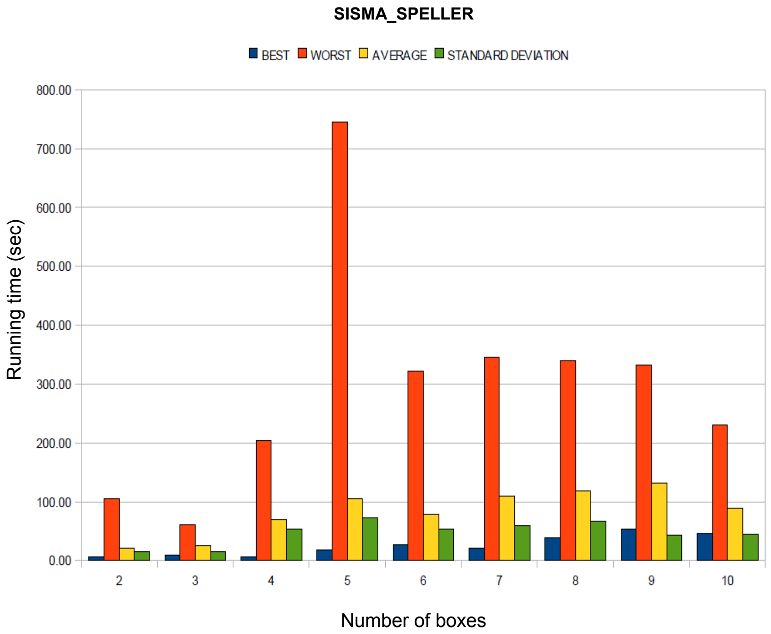

Figure 6: Best, Worst, Average runtimes and (in seconds) standard deviation for SISMA_SPELLER. Average runtimes and standard deviations have been computed omitting best and worst run.

running time of RISOTTO varies considerably according to the position of the $(14, 4)$ box (from 1 to 50 minutes): when this is in the first or second position, RISOTTO results much slower (and much slower than SISMA_SMILE). When moving the $(14, 4)$ box to increasing order positions RISOTTO becomes faster and outperforms SISMA_SMILE.

The explanation of this phenomenon comes by observing that, while in the 2-stage approach simple motifs are always searched into the whole input sequences, the search space of simple motifs explored by RISOTTO becomes narrower with box ordering position. In fact, the simple motifs for the i-th box are searched on the factor tree only in subtrees rooted at nodes where the occurrence-paths of $(i-1)$-prefixes end, that is in subsequences of the input sequences. However, when the $(14, 4)$ box is in first position, such search space reduction only involves simple motifs that are "easy" and thus the corresponding time saved (with respect to the 2-stage approach) is not significant.

The position of the $(14, 4)$ box is not the only factor influencing RISOTTO's running time. In fact, the mere combination of boxes according to different orderings may (and in general does) give rise to different numbers of structured motifs. In the present example, there are 4699 pairs of motifs $((14, 4)(12, 3))$ that satisfy both distance and quorum constraints, while there are 8525 pairs of motifs $((12, 3)(14, 4))$ satisfying the same constraints. This explains the running times reported in columns 1 and 2 for RISOTTO. Whatever the combinatorics, it is always the case that search spaces for simple motifs shrink as these occur "later" in a structured motif. In this example, the gain in running time due to search space reduction prevailed over any other negative combinatorial effect starting from position 3 of the hard box $(14, 4)$, where RISOTTO was definitely faster than SISMA_SMILE[2]. Observe, however, that the worst case scenario is once again adverse to RISOTTO, which takes about 50 min to solve the same instance for which SMILE requires less than 7 min.

[2]This phenomenon is much more evident in the supplementary set of test shown in Additional file 2, where very hard instances of the PMP are present.
